# Wheat miRNA TaemiR408 Acts as an Essential Mediator in Plant Tolerance to Pi Deprivation and Salt Stress via Modulating Stress-Associated Physiological Processes

**DOI:** 10.3389/fpls.2018.00499

**Published:** 2018-04-18

**Authors:** Qianqian Bai, Xiaoying Wang, Xi Chen, Guiqing Shi, Zhipeng Liu, Chengjin Guo, Kai Xiao

**Affiliations:** Key Laboratory of Crop Growth Regulation of Hebei Province, College of Agronomy, Agricultural University of Hebei, Baoding, China

**Keywords:** wheat (*Triticum aestivum* L.), miRNA member, Pi starvation, salt stress, plant growth, Pi acquisition, abiscisic acid signaling

## Abstract

MicroRNAs (miRNA) families act as critical regulators for plant growth, development, and responses to abiotic stresses. In this study, we characterized TaemiR408, a miRNA family member of wheat (*Triticum aestivum*), for the role in mediating plant responses to Pi starvation and salt stress. TaemiR408 targets six genes that encode proteins involving biochemical metabolism, microtubule organization, and signaling transduction. 5′- and 3′-RACE analyses confirmed the mRNA cleavage of target genes mediated by this wheat miRNA. TaemiR408 showed induced expression patterns upon Pi starvation and salt stress and whose upregulated expression was gradually repressed by the normal recovery treatments. The target genes of TaemiR408 exhibited reverse expression patterns to this miRNA, whose transcripts were downregulated under Pi starvation and salt stress and the reduced expression was recovered by the followed normal condition. These results suggest the regulation of the target genes under TaemiR408 through a cleavage mechanism. Tobacco lines with TaemiR408 overexpression exhibited enhanced stress tolerance, showing improved phenotype, biomass, and photosynthesis behavior compared with wild type under both Pi starvation and salt treatments, which closely associate increased P accumulation upon Pi deprivation and elevated osmolytes under salt stress, respectively. Phosphate transporter (PT) gene *NtPT2* displays upregulated transcripts in the Pi-deprived TaemiR408 overexpressors; knockdown of this PT gene reduces Pi acquisition under low-Pi stress, confirming its role in improving plant Pi taken up. Likewise, *NtPYL2* and *NtSAPK3*, genes encoding abscisic acid (ABA) receptor and SnRK2 protein, respectively, exhibited upregulated transcripts in salt-challenged TaemiR408 overexpressors; knockdown of them caused deteriorated growth and lowered osmolytes amounts of plants upon salt treatment. Thus, TaemiR408 is crucial for plant adaptations to Pi starvation and salt stress through regulating Pi acquisition under low-Pi stress and remodel ABA signaling pathway and osmoprotects biosynthesis under salt stress.

## Introduction

Phosphorus (P) deficiency and salt stress are two of the pronouncing abiotic stresses negatively regulating plant growth, development, and crop productivity. A line of evidence has suggested that plants cope with Pi starvation to sometimes cross-talk with the salt stress tolerance (Baek et al., 2016). To cope with low-Pi stress, plants have evolved a variety of adaptive mechanisms, such as modulation of root system architecture (RSA) establishment, enhancement of Pi acquisition activity, and elevation of secretion of organic acids and phosphatases (Raghothama, 1999; Poirier and Bucher, 2002; Yuan and Liu, 2008; Péret et al., 2011). Likewise, plants have also established a subset of pathways to withstand salt stress, through regulating osmolyte biosynthesis, stomota movement, cellular structure, and developmental plasticity via an abscisic acid (ABA)-dependent cascade (Giraudat, 1995; Zhu, 2002; Raghavendra et al., 2010). Currently, a suite of signaling components in the ABA-mediated pathway, including ABA receptors, kinases, transcription factors, and distinct ubiquitin ligases, has been functionally characterized (Hauser et al., 2011). Further elucidating the molecular mechanisms underlying plant responses to Pi starvation and salt stress are helpful in engineering crop cultivars with improved tolerance to these adverse stressors.

MicroRNAs (miRNAs) are 21 to 24 nt-long endogenous non-coding RNAs that widely mediate growth, development, and adaptive response to abiotic stresses via regulating target genes at the posttranscriptional or translation level (Bonnet et al., 2006; Mallory and Vaucheret, 2006; Sunkar et al., 2012; Ci et al., 2015; Sun et al., 2015). Numerous miRNA families identified have been confirmed to impact on plant responses to nutrient deprivation and salt stress (Jones-Rhoades and Bartel, 2004; Fujii et al., 2005; Chiou et al., 2006; Yamasaki et al., 2007; Liang et al., 2010; Barciszewska-Pacak et al., 2015; Khaksefidi et al., 2015; Kumar et al., 2017). Upon Pi starvation, miR156, miR399, miR778, miR827, and miR2211 of Arabidopsis (*Arabidopsis thaliana*) display an upregulated expression pattern (Fujii et al., 2005). Of which, miR2111 mediates Pi starvation response to be associated with its regulation on target gene encoding a Kelch repeat-containing F-box protein (Hsieh et al., 2009). miR827 mediates transduction of both Pi starvation and N deprivation signaling, acting as a central regulator in mediating response to both nutrients via negatively regulating an ubiquitin E3 ligase gene involving anthocyanin biosynthesis (Hsieh et al., 2009; Pant et al., 2009). Characterization on miR399 in more detail has elucidated the mechanism underlying the miRNA/target module-mediated internal Pi homeostasis; miR399 regulates Pi starvation response through its role in modifying the target *PHO2* transcripts, which further impact Pi uptake and internal P translocation across tissues via transcriptionally regulation of the PT genes (Fujii et al., 2005; Chiou et al., 2006; Pant et al., 2009). Thus, distinct miRNA members are Pi-starvation response and involved in taken up and homeostasis of Pi through the action of the miRNA/target modules. Thus far, although the genomic organization of plant miRNAs is much clear, the mechanisms underlying miRNAs-mediated response to external Pi are necessary for further investigation given the complicate regulatory modes mediated by the miRNA members.

Dissection of the salt stress-associated regulatory networks is helpful for further understanding the complex mechanism underlying salt adaptation of plants. A line of evidence has validated the critical roles of the miRNAs in plant salt response. A set of miRNA members, such as miR156, miR159, miR167, miR168, miR171, miR319, and miR396 of Arabidopsis, exhibit altered expression levels upon salt (Yu et al., 2005; Ding et al., 2009). miR399f, a Pi-starvation response member, also confers enhanced salt tolerance via transcriptionally regulating target genes *ABF3* and *CSP41b* (Baek et al., 2016). miR394 mediates plant response to salt and drought stresses in an ABA-dependent manner, whose interaction with target gene *LCR* transcriptionally modulates a suite of the ABA- and stress-responsive genes, such as *ABI3*, *ABI4*, *ABI5*, *ABF3*, and *ABF4* (Song et al., 2013). miR528 regulates plant salt response through modulation of diverse physiological processes associated with water retention, cell membrane integrity, chlorophyll content, and potassium homeostasis, and activities of catalase and ascorbic acid oxidase (Yuan et al., 2015). These findings provide novel insights into plant salt stress response underlying the miRNA/target action modules.

miR408 is a conserved miRNA family member across diverse plant species. Previous investigations have revealed its transcriptional response to stresses of copper deprivation (Ma et al., 2015), nitrogen and sulfur deficiency (Liang et al., 2015), and the critical role in regulating photosynthesis and grain yield (Zhang et al., 2017), heading time (Zhao et al., 2016), iron uptake (Paul et al., 2016), and drought tolerance (Hajyzadeh et al., 2015). These findings suggest that miR408 acts as a crucial regulator in multiple biological processes. To date, although a line of investigations in wheat on identifying miRNA target genes (Sun et al., 2014), characterizing miRNA/target expression patterns upon abiotic stresses (Zhao et al., 2013; Wang et al., 2014; Sinha et al., 2015; Bakhshi et al., 2017), and evaluating miRNA-mediated plant adaptation to stressors, such as Pi starvation (Ouyang et al., 2016) and N deprivation (Gao et al., 2016), has been performed, the function of wheat miR408 (TaemiR408) remains largely unknown. In this study, we characterized the role of TaemiR408 in mediating plant adaptation to Pi starvation and salt stress. Our results indicate that this miRNA plays crucial roles in regulating plant low-Pi stress and salt adaptations through modulating stress-associated physiological processes.

## Materials and Methods

### Characterizing the Conserved Nature of TaemiR408

Based on expression analysis for the miRNA families of wheat deposited in miRNA database^1^, a suite of the miRNA members were shown to be responses to drought, salt, and deprivations of nitrogen (N), and phosphate (Pi) (our unpublished data). Among these, TaemiR408 (accession number MI0006177) showed induced expression upon both Pi starvation and salt stress, suggesting the miRNA-mediated plant responses to these stressors. To understand whether miR408 is evolutionarily conserved across plant species, we amplified the TaemiR408 homolog of tobacco, a model eudicot plant species frequently used in genetic analysis, based on reverse transcriptase-polymerase chain reaction (RT-PCR) using the TaemiR408 specific primers (**Supplementary Table S1**). Root cDNA of tobacco (*Nicotiana tabacum* cv. Wisconsin 35) was acted as templates.

### Predicting the Target Genes of TaemiR408

miRNA-mediated biological processes closely associate the miRNA regulation on target genes at posttranscriptional or translation level. To define the target genes of TaemiR408, an online tool referred to as psRNATarget (Plant microRNA Potential Target Finder^2^) was adopted to predict the genes putatively interacted by TaemiR408. As suggested, the mature TaemiR408 sequence was subjected to scanning against two of wheat cDNA databases, including (i) *Triticum aestivum* (bread wheat), transcript, cDNA library, TGACv1 and (ii) *Triticum aestivum* (wheat), DFCI Gene Index (TAG), version 12, released by 2010-04-18. Biological functions of the six target genes identified were defined based on gene BLASTn search analysis in NCBI.

### Expression Analysis of TaemiR408, miR408 Tobacco Homolog, and Target Genes

The expression patterns of TaemiR408, miR408 tobacco homolog (referred to as NtMIR408 hereafter), and the target genes of TaemiR408 upon Pi starvation and salt stress were evaluated. With this aim, wheat (cv. Shiyou 20) and tobacco (cv. Wisconsin 35) seedlings were cultured normally in standard Murashige and Skoog (MS) solution to the fourth leaf stage in a growth chamber under follow condition: photoperiod of 12 h/12 h (day/night) with 300 μmolE⋅m^-2^⋅s^-1^ light intensity during light phase, temperature of 28°C/23°C (day/night), and a relative humidity from 65 to 70%. They were transferred in the modified MS solutions either containing reduced Pi (0.012 mM Pi) or supplemented with NaCl (200 mM) for stress treatments and followed recovery treatments, which were established by transferring the 48 h-stressed seedlings again to standard MS solution. At time points of 0 h (before treatment), 6, 12, 24, and 48 h after stress treatment, and 6, 12, 24, and 48 h after recovery treatment, root tissues were collected and subjected to expression evaluation for TaemiR408, NtMIR408, and the target genes based on qRT-PCR performed as previously described (Guo et al., 2013). Briefly, total RNA from roots was extracted by TRIzol reagents (Invitrogen, United States). After treatment with RNase-free DNase (TaKaRA, Dalian, China) to avoid genomic DNA contamination, the total RNA (2 μg) was subjected to synthesis for the first-strand cDNA with RT-AMV transcriptase (TaKaRa, Dalian, China) in 20 μL reaction volume using oligo (dT)18 at 42°C for 30 min, as suggested. qRT-PCR analysis was performed in a total volume of 25 μL containing 12.5 μL of SYBR Premix ExTaqTM (TaKaRa, Dalian, China), 0.5 μL of forward and reverse primers, 1 μL cDNA and 10.5 μL nuclease-free water. Transcripts of the miRNA members and the target genes were calculated based on the by 2^-ΔΔ*CT*^ method using wheat *Tatubulin* and tobacco *Nttubulin* as an internal control. The gene-specific primers used for qRT-PCR analysis are shown in **Supplementary Table S1**.

### Identification of the Target Cleavage Characterization by TaemiR408

To verify the target genes acted by TaemiR408, we analyzed the cleavage products of them based on Poly (A) polymerase -mediated 3′ rapid amplification of cDNA ends (PPM-RACE) and RNA ligase-mediated 5′ rapid amplification of cDNA ends (RLM-RACE) as previously reported (Song et al., 2012). Briefly, total RNA of wheat (cv. Shiyou 20) roots at various time points including at normal growth (0 h, CTR), 48 h after Pi starvation, and 48 h after Pi normal recovery was polyadenylated at 37°C for 60 min in a 50 μl reaction mixture (containing 5 μg of total RNA, 1 mM ATP, 2.5 mM MgCl_2_, and 8 U poly (A) polymerase) (Ambion, United States). They were then subjected to ligation to a 5′ adapter (5′-CGACUGGAGCACGAGGACACUGACAUGGACUGAAGGAGUAGAAA-3′) using T4 RNA ligase (TaKaRa, Japan). PPM-RACE and RLM-RACE were performed using the GeneRacer kit (Invitrogen). The GeneRacer Nested Primer and gene-specific primers for PPM-RACE and RLM-RACE reactions are shown in **Supplementary Table S1**. mRNA cleavage products were detected based on qRT-PCR performed similarly as previously described (Guo et al., 2013).

### Establishment of Transgenic Tobacco Lines Overexpressing TaemiR408

A transgene analysis was performed to characterize whether TaemiR408 is involved in the mediation of plant responses to Pi starvation and salt stresses. Given the conserved nature of miR408 across wheat and tobacco as described above, we transformed TaemiR408 into tobacco, due to its genetic transformation conveniently relative to that of wheat. To this end, RT-PCR was performed to amplify the TaemiR408 precursor sequence using specific primers (**Supplementary Table S1**), the amplified product was then inserted into the *Nco*I/*BstE*II restriction sites in binary vector pCAMBIA3301 under the control of CaMV35S promoter. Then the expression cassette was integrated into *Agrobacterium tumefaciens* strain EHA105 using conventional heat shock approach. Genetic transformation of tobacco (cv. Wisconsin 35) using EHA105 transformants and further generation of tobacco lines with TaemiR408 overexpression were conducted as described previously (Sun et al., 2012). The TaemiR408 transcripts in transgenic lines were evaluated based on qRT-PCR.

### Assays of Growth Features, Biomass, P-associated Traits, and Osmolyte Amounts and Target Gene Expression in Transgenics

Two T3 lines showing more TaemiR408 transcripts (**Supplementary Figure S2A**) and one copy insertion of target in genome (**Supplementary Figure S2B**), OE2 and OE3, were selected to characterize the TaemiR408 function in mediating responses to Pi starvation and salt stress. With this aim, 10-day-old evenly seedlings of the transgenic lines and wild type (WT) were cultured in plastic pots filled by vermiculite and supplied by standard MS solution (sufficient-Pi, 1.2 mM Pi) or modified MS solution containing reduced Pi (deficient-Pi, 0.05 mM Pi) for Pi starvation treatment, and with standard MS solution (control) or modified MS solution supplemented with salt (200 mM NaCl) for salt treatment. Three weeks later, the transgenic and WT plants were subjected to evaluation of phenotypes, biomass, P-associated traits, and osmolyte contents. Of these, the phenotypes were recorded based on images taken by a digital camera; biomass were obtained after oven drying; P concentrations and P amounts were assessed as described previously (Guo et al., 2011); contents of osmolytes (i.e., proline and soluble sugar) were analyzed as reported (Du et al., 2013). In addition, to understand expression patterns of the target genes in transgenics, we searched the *N. tabucum* cDNA database [DFCI Gene Index (NTGI), version 7] and identified three TaemiR408 target homologs, including *NtBCP* (TC142445), *NtKRP* (FS391808), and *NtAMP* (TC145853), using the online tool psRNATarget (Plant microRNA Potential Target Finder^3^). The expression patterns of these target genes in Pi starvation- and salt stress-challenged transgenic lines (OE2 and OE3) and wild type (WT) were assessed based on qRT-PCR using gene specific primers (**Supplementary Table S1**).

### Assays of Photosynthetic Parameters

Photosynthesis modified by internal and environmental cues impacts greatly on plant growth, development, and the response of plants to abiotic stresses, which causes reduction on dry mass production and yield potential (Gu et al., 2017). To address if the TaemiR408-modified plant biomass under Pi starvation and salt stress associate with the modulation of miRNA on photosynthesis behaviors, three parameters effectively reflecting photosynthetic function of leaves, including photosynthetic rate (Pn), PSII efficiency (ΦPSII), and non-photochemical quenching (NPQ), were investigated in the upper leaves of transgenic and WT plants after the Pi starvation and salt treatments. The parameters were assessed as described previously (Guo et al., 2013).

### Assays of Expression Patterns of Tobacco PT Genes and Functional Analysis of *NtPT2*

The Pi taken up of plants from growth media as well as internal P translocation across tissues are mediated by the membrane-bound phosphate transporters (PTs), during which uses ATP as the driving power (Mudge et al., 2002; Shin et al., 2004). The improved Pi accumulation in TaemiR408 overexpressors under Pi starvation prompted us to characterize the expression patterns of the tobacco PT genes. To this end, a suite of tobacco PT genes, including *NtPT* (DI040486), and *NtPT1* to *NtPT5* (AB020061, AF156696, AB042950, AB042951 and AB042956, respectively) was subjected to expression evaluation in the Pi-deprived transgenic lines and WT based on qRT-PCR using gene specific primers (**Supplementary Table S1**), with internal reference *Nttubulin* for expression normalization.

PT gene expression analysis as above revealed that one of them, *NtPT2*, exhibited significantly upregulated expression in transgenic lines relative to WT, suggesting its potential in the regulation of Pi acquisition in Pi-deprived transgenic plants. To functionally characterize its role in mediating Pi taken up, transgenic lines with knockdown of this PT gene were generated. With this aim, RT-PCR was performed to amplify the open reading frame (ORF) of *NtPT2* in antisense orientation using specific primers (**Supplementary Table S1**), the amplified product was inserted into *Nco*I/*BstE*II restriction sites of binary vector pCAMBIA3301 downstream the CaMV35S promoter. Integration of the expression cassette into EHA105 and transformation of *NtPT2* into tobacco were performed to be similar in establishment of the TaemiR408 overexpressors. *NtPT2* transcripts in lines with knockdown of this PT gene were evaluated based on qRT-PCR. Two lines with much less target transcripts at T3 generation (Anti-PT2-2 and Anti-PT2-5) were selected for further functional analysis. For this, 10-day-old transgenic and WT seedlings were vertically cultured on agar MS medium (sufficient-Pi, 1.2 mM) and modified MS nutrients with reduced Pi (deficient-Pi, 0.05 mM) for Pi normal and starvation treatments, respectively. Two weeks later, phenotypes, biomass, and P-associated traits in transgenic lines and WT were evaluated performed to be similar in assessing these traits in OE2 and OE3.

### Assays of Expression Patterns of Tobacco ABA Signaling Genes and Transgene Analysis of *NtABR2* and *NtSPK1*

ABA-dependent signaling pathways are closely associated with plant response to the abiotic stresses, including water deficit and salt stress (Fujii and Zhu, 2009). Of which, the ABA receptors and SnRK2 family proteins act as critical mediators in transduction the ABA signaling (Ma et al., 2009; Park et al., 2009). To characterize if the TaemiR408-mediated salt response is related to the ABA signaling, a suite of ABA receptor and SnRK2 encoding genes released in the NCBI GenBank database, including seven ABA receptor genes (i.e., *NtPYR1*, *NtPYL2*, *NtPYL4*, *NtPYL8*, *NtPYL9*, *NtPYL11*, and *NtPYL12*) and ten SnRK2 kinase genes (i.e., *NtSAPK1*, *NtSAPK2, NtSAPK2;1, NtSAPK2A*, *NtSAPK2A;1*, *NtSAPK2E*, *NtSAPK2E;1*, *NtSAPK2I*, *NtSAPK3*, and *NtSAPK7*), was subjected to expression evaluation in the salt-challenged TaemiR408 overexpressors and WT. qRT-PCR was performed to understand the transcripts abundance of the ABA receptor and SnRK2 family genes, in which, *Nttubulin* was used as internal reference. Accession numbers and specific primers for these ABA signaling genes are listed in **Supplementary Table S1**.

Expression analysis revealed that *NtABR2* and *NtSAPK3* are significantly upregulated in the TaemiR408 overexpressors after salt stress, suggesting their potential in mediating salt stress response. Therefore, transgenic lines with knockdown of them were generated to define their function in mediating salt tolerance. With this purpose, RT-PCR was performed to amplify the ORFs of *NtABR2* and *NtSAPK3* in antisense orientation using specific primers (**Supplementary Table S1**), the ORFs were then inserted into *Nco*I/*BstE*II restriction sites of binary vector pCAMBIA3301 under the control of CaMV35S promoter. Transgenic lines were generated similarly in establishment of the TaemiR408 overexpressors as aforementioned. The T3 lines Anti-PYL2-2 and Anti-PYL2-3 for *NtABR2* and Anti-SAPK3-3 and NtSAPK3-4 for *NtSAPK3* were subjected to salt stress treatment by vertically culturing the evenly 7-day-old seedlings on agar media supplemented without or with 200 mM NaCl. Two weeks after treatments, phenotypes, biomass, and contents of proline and soluble sugar in these lines were assayed to be similar in assessment of those in TaemiR408 overexpression lines.

### Statistics Analysis

Averages of gene expression levels in qRT-PCR analysis, plant biomass, photosynthesis parameters, P concentrations and P accumulative amounts, proline contents, and soluble sugar contents in the transgenic lines and WT were derived from the results of four replicates. Standard errors of averages and significant differences were analyzed using the Statistical Analysis System software (SAS Corporation, Cory, NC, United States).

## Results

### miR408 Shows Conserved Nature Between Wheat and the Eudicot Tobacco

The mature sequence of TaemiR408 is 21 nt-long in length (5′-cugcacugccucuucccuggc-3′), which is situated in a 187 nt-long precursor. **Supplementary Figure S1A** shows the secondary stem-loop structure established by the TaemiR408 precursor. Given that most miRNAs share conserved nature across eukaryotes, we addressed if miR408 is also present in both monocot and eudicot by identifying the miRNA paralog in tobacco. Sequencing analysis on the RT-PCR product derived from tobacco (cv. Wisconsin 35) revealed an identical precursor of TaemiR408 in *N. tabacum* (designated as NtMIR408) (**Supplementary Figure S1B**). Our amplified NtMIR408 is not same as the miR408 member deposited in *N. tabacum* miRNA database (Accession No. MI0021410), suggesting that miR408 is conserved and the tobacco miR408 family consists of a set of members.

### TaemiR408 Targets Six Genes Functional in Various Biological Processes

**FIGURE 1:**
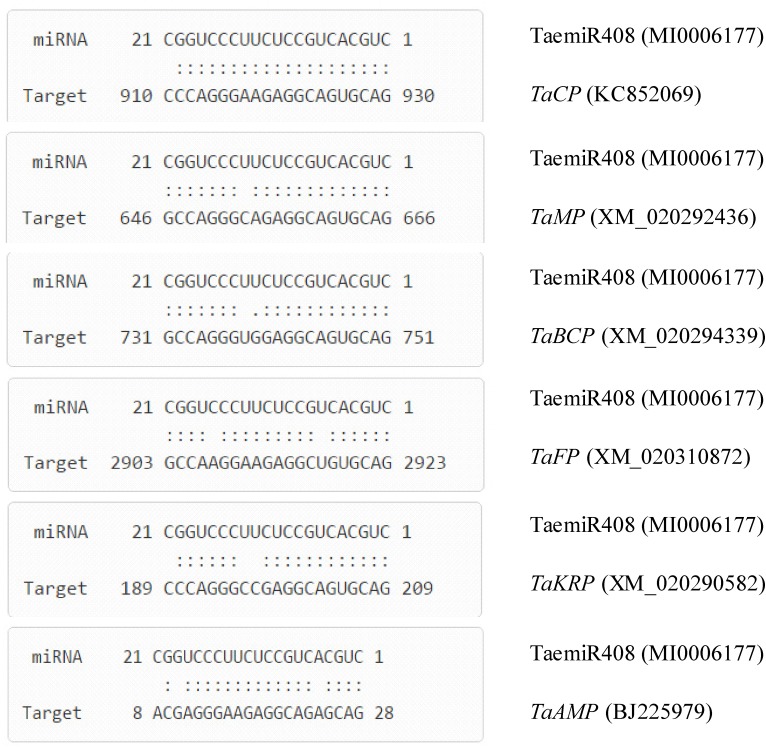
Base pairing characterization between TaemiR408 and its target genes. *TaCP* (wheat chemocyanin encoding gene), *TaMP* (wheat mavicyanin encoding gene), *TaBCP* (wheat blue copper protein encoding gene), *TaFP* (wheat filament encoding gene), *TaKRP* (wheat F-box/kelch-repeat protein encoding gene), and *TaAMP* (wheat AMP-binding protein encoding gene), six genes that are interacted by TaemiR408.

Based on running an online tool, the genes putatively targeted by TaemiR408 were predicted. Results indicated that totally six genes were interacted by this wheat miRNA, including five from wheat cDNA database and one from expressed sequence tag (EST) database. The target genes from the cDNA database were as follows: TRIAE_CS42_1BTRIAE_CS42_5DL_TGACv1_433117_AA1402600, a gene encoding chemocyanin putatively functional in biochemical metabolism (*TaCP*, KC852069); TRIAE_CS42_5AL_TGACv1_379889_AA1257020, a gene coding for mavicyanin functioning in primary biochemical process (*TaMP*, XM_020292436); TRIAE_CS42_7AS_TGACv1_571397_AA1847350, a gene encoding blue copper protein involving secondary biochemical metabolism (*TaBCP*, XM_020294339); TRIAE_CS42_U_TGACv1_641170_AA2087300, a gene for filament involving the cellular microtubule organization (*TaFP*, XM_020310872); and TRIAE_CS42_6BS_TGACv1_513906_AA1651810, a gene for F-box/kelch-repeat protein functioning in protein-protein interaction (*TaKRP*, XM_020290582). The target gene from the EST database encodes an AMP-binding protein involving the binding of cyclic AMP (*TaABP*, BJ225979). **Figure 1** shows the base pairing characterization between TaemiR408 and the target genes. BLASTn analysis for the TaemiR408 target genes suggested that they are categorized into diverse function families, including three involved in biochemical metabolism (i.e., *TaCP TaMP*, and *TaBCP*), two in signaling transduction (*TaKRP* and *TaABP*), and one in microtubule organization (*TaFP*). Thus, TaemiR408 establishes putative action module(s) with the target genes and exerts distinct biological functions in plants.

### miR408 and the Target Genes Are Responses to Pi Starvation and Salt Stress

**FIGURE 2:**
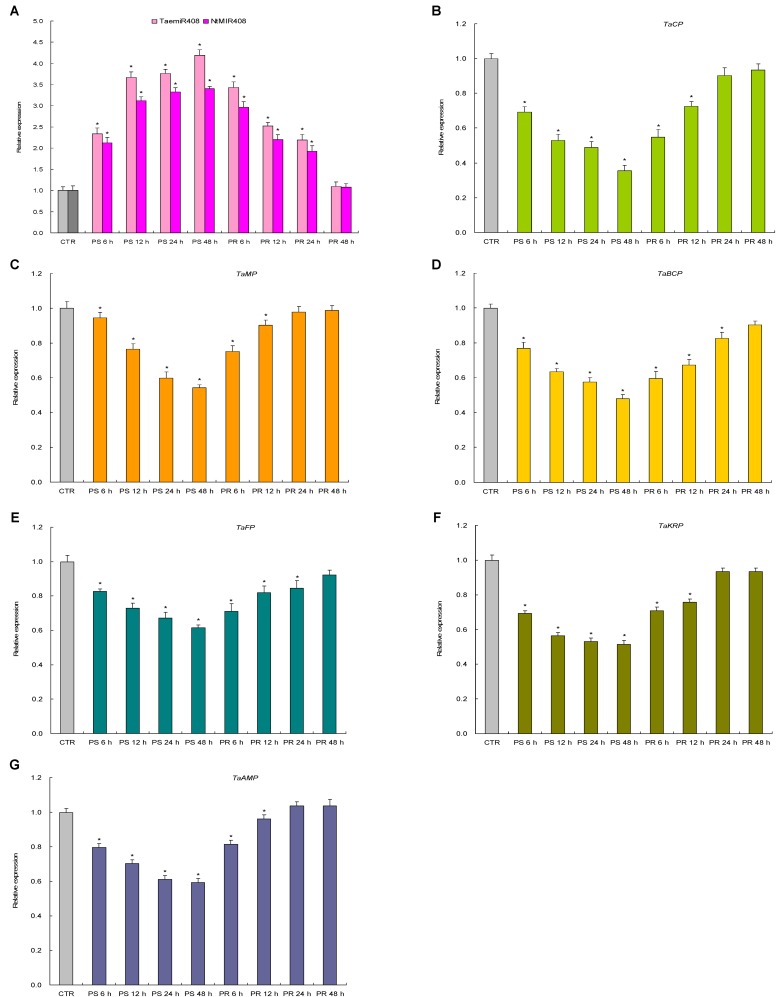
The expression patterns of TaemiR408, its tobacco paralog NtMIR408, and the target genes of TaemiR408 upon Pi starvation and Pi recovery treatments. **(A)** Expression patterns of TaemiR408 and NtMIR408; **(B)** expression patterns of *TaCP*; **(C)** expression patterns of *TaMP*; **(D)** expression patterns of *TaBCP*; **(E)** expression patterns of *TaFP*; **(F)** expression patterns of *TaKRP*, **(G)** expression patterns of *TaAMP.* Data are normalized by internal standards and shown by average plus standard error. CTR, control (before Pi starvation treatment). PS 6 h, PS 12 h, PS 24 h, and PS 48 h, time points after Pi starvation treatment. PR 6 h, PR 12 h, PR 24 h, and PR 48 h, time points after Pi recovery treatment. In **(A–G)**, data are shown by average plus standard error and ^∗^ indicates to be statistically significant compared with CTR (*P* < 0.05).

**FIGURE 3:**
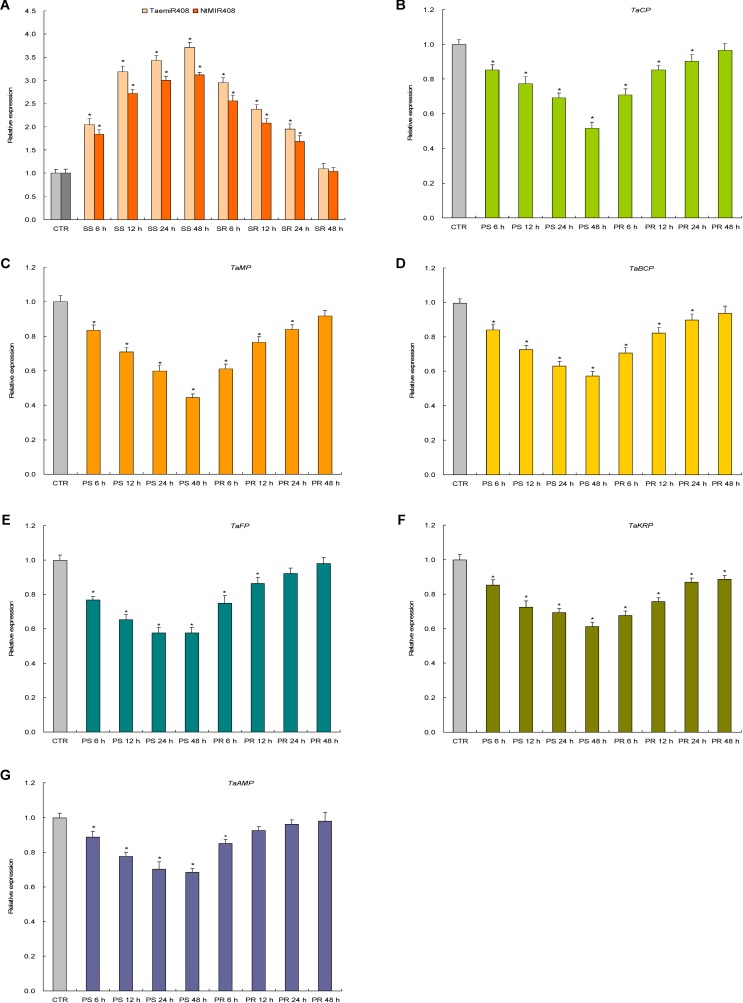
The expression patterns of TaemiR408, its tobacco paralog NtMIR408, and the target genes of TaemiR408 upon salt stress and normal recovery treatments. **(A)** Expression patterns of TaemiR408 and NtMIR408; **(B)** expression patterns of *TaCP*; **(C)** expression patterns of *TaMP*; **(D)** expression patterns of *TaBCP*; **(E)** expression patterns of *TaFP*; **(F)** expression patterns of *TaKRP*; **(G)** expression patterns of *TaABP.* Data are normalized by internal standards and shown by average plus standard error. CTR, control (before salt treatment). PS 6 h, PS 12 h, PS 24 h, and PS 48 h, time points after salt treatment. PR 6 h, PR 12 h, PR 24 h, and PR 48 h, time points after normal recovery treatment. In **(A–G)**, data are shown by average plus standard error and ^∗^ indicates to be statistically significant compared with CTR (*P* < 0.05).

Expression patterns of TaemiR408, NtMIR408, and the target genes of TaemiR408 upon Pi starvation and salt stress were investigated in more detail. Results indicated a similar expression pattern for TaemiR408 and NtMIR408 under the stresses as well as upon recovery treatments; the two miRNAs both showed upregulated transcripts abundance over 48 h regimen of the stressors and whose elevated expression was downregulated along with 48 h regimen of the normal recoveries (**Figures 2A**, **3A**). All of the target genes displayed reverse expression patterns to miRNAs upon Pi starvation and Pi recovery treatments as well as upon salt stress and salt recovery treatments; they showed a downregulated expression pattern over 48 h regimen of the stressors and whose repressed transcripts under stresses were gradually restored along with 48 h regimen of the recoveries, albeit that the expression amplitudes of them in response to these stressors are different (**Figures 2B–G**, **3B–G**). Therefore, TaemiR408 regulates the target genes post-transcriptionally and establishes putative miRNA/target modules that mediate plant Pi starvation and salt responses.

### mRNA Fragments of the Target Genes Are Cleaved by TaemiR408

**FIGURE 4:**
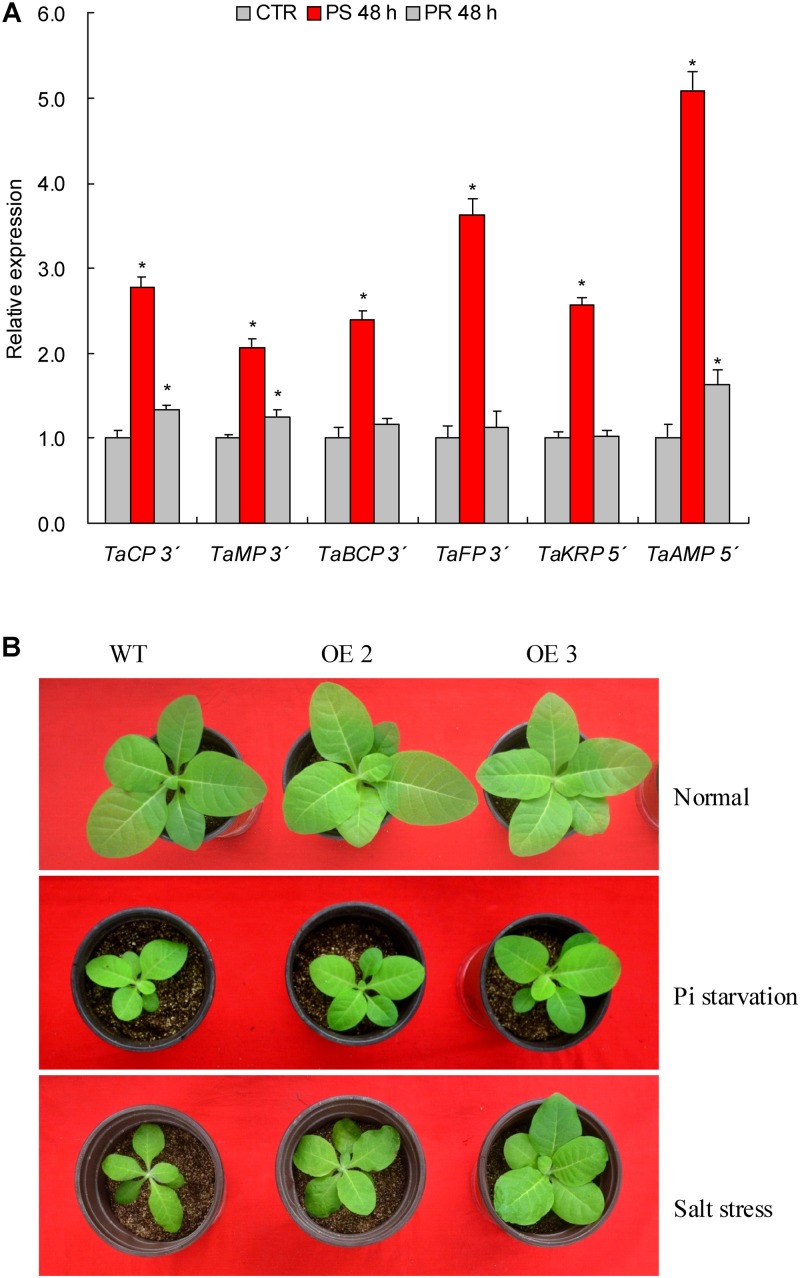
The 5′- and 3′-RACE results of target genes and phenotypes of TaemiR408 overexpression lines under the Pi starvation and salt stress treatments. (A) The 5′- and 3′-RACE results of the target genes. **(B)** The phenotypes of TaemiR408 overexpression lines under the Pi starvation and salt stress treatments. In **(A)**, *TaCP*, *TaMP*, *TaBCP*, *TaFP*, *TaKRP* and *TaABP*, six genes that are targeted by TaemiR408. CTR, control (before Pi starvation treatment). PS 48 h, PR 48 h, the time points of 48 h after Pi starvation and 48 h after Pi normal recovery treatments, respectively. Data are shown by average plus standard error and ^∗^ indicates to be statistically significant compared with CTR (*P* < 0.05). In **(B)**, OE2 and OE3, two lines with TaemiR408 overexpression; WT, wild type.

To experimentally verify the target genes, PPM-RACE and RLM- RACE that effectively detect the 3′ and 5′ end fragments of the cleaved products after miRNA mediation, respectively, were performed. Among the six target genes (i.e., *TaCP*, *TaMP*, *TaBCP*, *TaFP*, *TaKRP*, and *TaAMP*) that showed converse expression patterns to TaemiR408 upon Pi starvation and salt stress, we detected the 3′ end products of *TaCP*, *TaMP*, *TaBCP*, and *TaFP* based on PPM-RACE and the 5′ end products of *TaKRP* and *TaAMP* by RLM- RACE in the roots of normal growth (0 h, CTR), 48 h after Pi starvation, and 48 h after Pi recovery. At the assayed time points, the cleavage products of the target genes are all consistent with the miRNA transcripts abundance (**Figures 2A**, **4A**). These results confirm the regulation of these target genes under TaemiR408 at the posttranscriptional level.

### TaemiR408 Confers Improved Growth and Pi Acquisition of Plants Upon Pi Starvation

**FIGURE 5:**
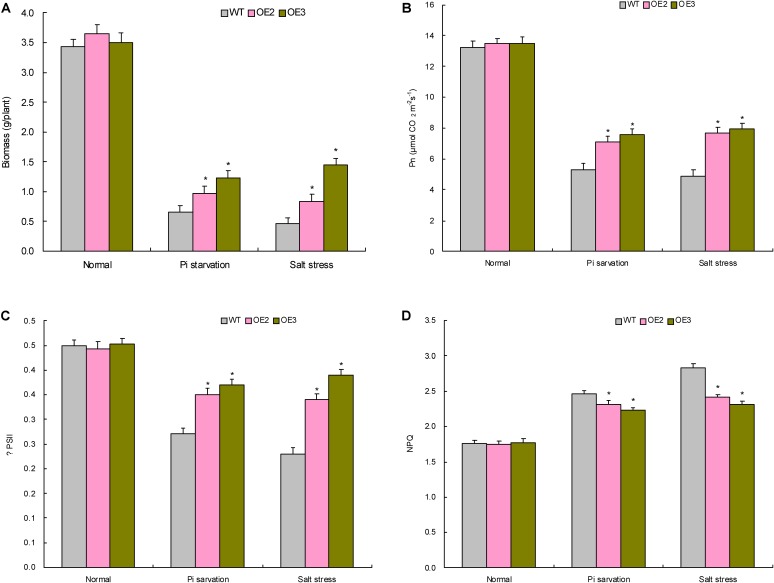
The biomass and photosynthesis parameters of the transgenic lines with TaemiR408 overexpression under the Pi starvation and salt stress treatments. **(A)** Biomass; **(B)** Pn; **(C)** ΨPSII; **(D)** NPQ. OE2 and OE3, two lines with TaemiR408 overexpression; WT, wild type. Data are shown by average plus standard error and ^∗^ indicates to be statistically significant compared with WT (*P* < 0.05).

**FIGURE 6:**
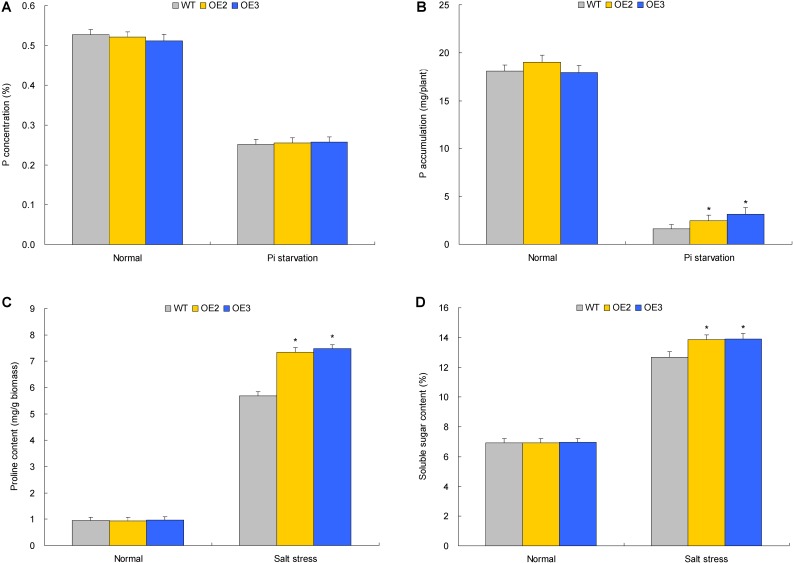
The P concentrations, P accumulation, proline contents, and the soluble sugar contents in transgenic lines with TaemiR408 overexpression under the Pi starvation and salt stress treatments. **(A)** P concentration; **(B)** P accumulation; **(C)** proline content; **(D)** soluble sugar content. OE2 and OE3, two lines with TaemiR408 overexpression; WT, wild type. **(A,B)** Data sets shown are obtained under the Pi starvation treatment; **(C,D)** data sets shown are obtained under the salt stress treatment. Data in **(A–D)** are shown by average plus standard error and ^∗^ indicates to be statistically significant compared with WT (*P* < 0.05).

Two TaemiR408 overexpressors (OE2 and OE3) were cultured under contrasting Pi levels for miRNA functional analysis in mediating Pi starvation response. Under the sufficient-Pi condition, OE2 and OE3 exhibited comparable phenotypes (**Figure 4B**), biomass (**Figure 5A**), photosynthesis parameters (i.e., Pn, ΦPSII, and NPQ) (**Figures 5B–D**), P concentrations (**Figure 6A**), and P accumulative amounts (**Figure 6B**) with WT. In contrast, under the Pi-starvation stress, the transgenic lines displayed modified growth, P-associated traits, and photosynthetic parameters, showing improved phenotypes (**Figure 4B**), elevated biomass (**Figure 5A**), P accumulation (**Figure 6A**), Pn and ΦPSII, and decreased NPQ (**Figures 5B–D**) relative to WT, although similar Pi concentrations were observed in the Pi-deprived transgenic and WT plants (**Figure 6A**). Additionally, the miR408 target genes including *NtBCP*, *NtKRP*, and *NtAMP* showed lowered expression levels in the transgenic lines (OE2 and OE3) than in wild type under low-Pi stress (**Supplementary Figures S3A–C**). These results suggest the critical role of TaemiR408 in mediating Pi starvation tolerance through regulation of target genes at the posttranscriptional level.

### *NTPT2* Shows Modified Transcripts in Pi-Deprived Transgenic Lines and Contributes to Plant Pi Acquisition

**FIGURE 7:**
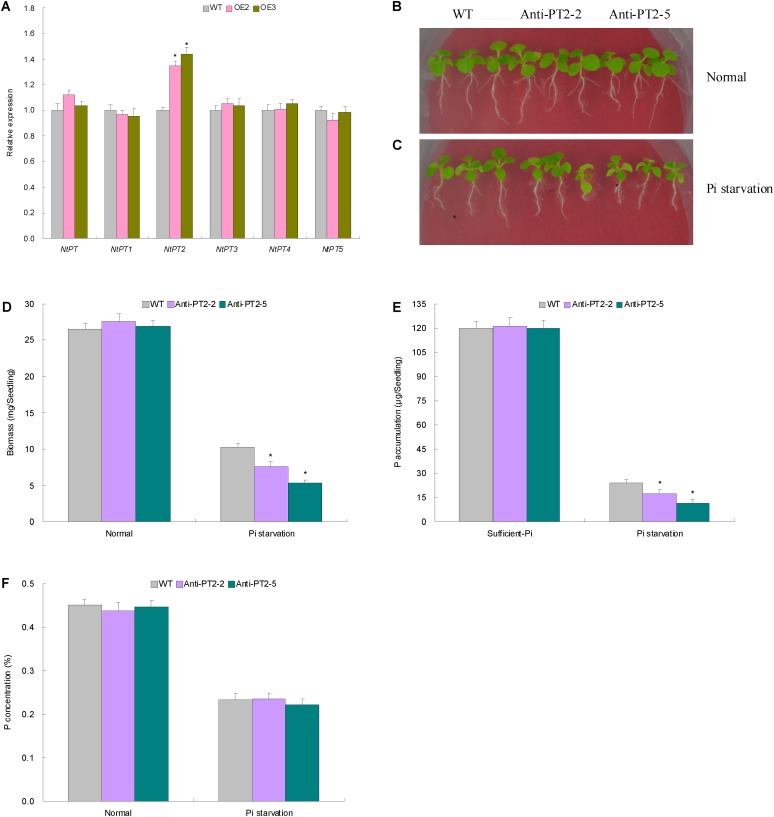
The expression patterns and the biological function results on the tobacco phosphate transporter (PT) genes. **(A)** The expression patterns of the tobacco PT genes in the Pi-deprived WT and lines with TaemiR408 overexpression. **(B)** The phenotypes in the lines with *NtPT2* knockdown expression under the normal condition; **(C)** the phenotypes in the lines with *NtPT2* knockdown under the Pi starvation treatment; **(D)** the biomass in the lines with *NtPT2* knockdown expression; **(E)** the P accumulation in the lines with *NtPT2* knockdown expression; **(F)** the P concentrations in the lines with *NtPT2* knockdown expression. In **(D–F)**, Anti-PT2-2 and Anti-PT2-5, two lines with *NtPT2* knockdown; WT, wild type. In **(A)**, and **(D–F)**, data are shown by average plus standard error and ^∗^ indicates to be statistically significant compared with WT (*P* < 0.05).

The more P amounts in Pi-deprived TaemiR408 overexpression lines suggested the contribution of an increased Pi acquisition capacity of root system to the miRNA-mediated low-Pi tolerance. To characterize the PT genes involving the elevated root Pi taken up in transgenic lines, six tobacco PT genes (i.e., *NtPT*, and *NtPT1* to *NtPT5*) were subjected to expression evaluation in the Pi-deprived transgenic and WT plants. Among them, *NtPT2* showed significantly upregulated expression in lines OE2 and OE3 compared with that in WT, which was contrast to other genes displaying unaltered expression among the Pi-deprived transgenic and WT plants (**Figure 7A**). This finding suggests the transcriptional regulation of *NtPT2* under TaemiR408.

Two lines with *NtPT2* knockdown (Anti-PT2-2 and Anti-PT2-5, **Supplementary Figure S4**) were cultured under two contrasting Pi conditions to characterize the PT role in mediating Pi acquisition. Under sufficient-Pi condition, no obvious variations were observed on phenotype, biomass, P concentration, and P accumulation among the transgenic lines and WT (**Figures 7B–F**). Under Pi starvation treatment, however, the lines with *NtPT2* knockdown exhibited deteriorated phenotypes (**Figure 7C**), and decreased biomass (**Figure 7D**) and P amounts (**Figure 7E**) relative to WT. *NtPT2* thus acts as a high-affinity PT gene and contributes to the TaemiR408-improved Pi accumulation.

### TaemiR408 Improves Growth and Osmolytes Accumulation of Plants Upon Salt Stress

Similar to the investigation for TaemiR408-mediated plant Pi starvation response as described above, OE 2 and OE 3, two TaemiR408 overexpressors, were cultured in vermiculite supplemented with NaCl (200 mM) to evaluate the miRNA-mediated salt tolerance. Under normal condition, the transgenic lines exhibited comparable phenotypes (**Figure 4**) and biomass (**Figure 5A**) with WT, which were comparable to those cultured under the sufficient-Pi condition as aforementioned. Additionally, no obvious variations were observed on photosynthesis parameters (i.e., Pn, ΦPSII, and NPQ) (**Figures 5B–D**) and contents of proline (**Figure 6C**) and soluble sugar (**Figure 6D**) in transgenic and WT plants. Under salt stress treatment, the TaemiR408 overexpression lines displayed improved phenotypes, biomass, elevated proline and soluble sugar contents, and modified photosynthesis parameters (i.e., increased Pn and ΦPSII and decreased NPQ) with respect to WT (**Figures 4**, **5A–D**, **6C,D**). Moreover, expression analysis revealed that *NtBCP*, *NtKRP*, and *NtAMP*, three target genes of miR408, exhibited reduced transcripts abundance in the transgenic lines (OE2 and OE3) than in the wild type plants under salt stress treatment (**Supplementary Figures S3A–C**). Therefore, TaemiR408 regulates the target genes at posttranscriptional level and plays important roles in mediating salt tolerance, which is associated with the miRNA-improved osmoregulatory capacity and photosynthesis function of plants upon salt stress.

### Expression Patterns of ABA Signaling Components and Roles of *NtPYL2* and *NtSAPK3* in Regulating Salt Tolerance

**FIGURE 8:**
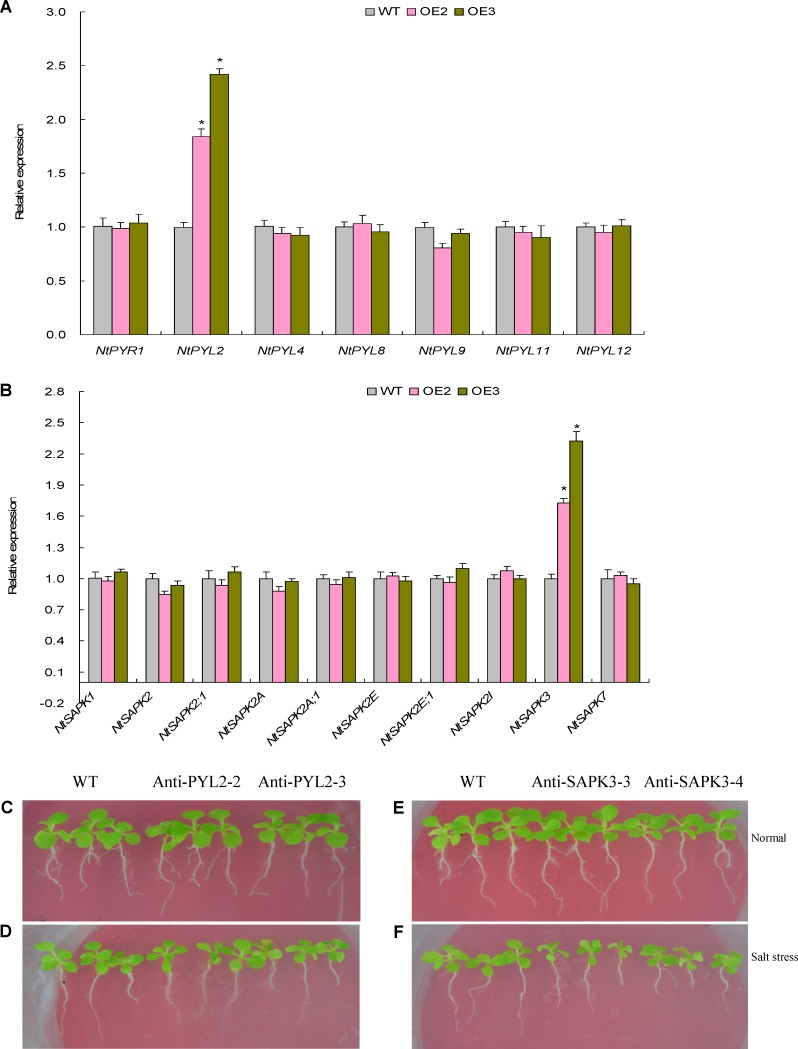
The expression patterns and functional characterization results on the tobacco ABA signaling genes. **(A)** The expression patterns of the ABA receptor genes; **(B)** the expression patterns of the SnRK2 family genes; **(C)** the phenotypes in the lines with *NtPYL2* knockdown expression; **(D)** the phenotypes in the lines with *NtSPAK3* knockdown expression. In **(A,B)**, data are shown by average plus standard error and ^∗^ indicates to be statistically significant compared with WT (*P* < 0.05). In **(C,D)**, Anti-PYL2-2 and Anti-PYL2-3, two lines with *NtPYL2* knockdown expression. In **(E,F)**, Anti-SAPK3-3 and Anti-SAPK3-4, two lines with *NtSAPK3* knockdown expression.

Abscisic acid is swiftly induced in the tissues of plants upon osmotic stresses, such as high salinity and drought, which further involves the plant response to above stressors through an ABA-dependent pathway (Fujii and Zhu, 2009). That the TaemiR408-mediated salt tolerance associates elevated osmolytes prompted us to investigate whether the ABA signaling pathway is modulated by this miRNA member. To address this, a large set of the ABA signaling component genes, including seven encoding ABA receptors and ten coding for SnRK2 family proteins, were subjected to expression evaluation in the salt-challenged TaemiR408 overexpression lines. Among the genes examined, an ABA receptor gene *NtPYL2* and a SnRK2 gene *NtSAPK3* displayed significantly upregulated expression in the transgenic lines relative to WT (**Figures 8A,B**). Other genes aside from these two behaved unaltered transcripts in the transgenic and WT plants (**Figures 8A,B**). These expression results suggest the transcriptional regulation of *NtPYL2* and *NtSAPK3* under TaemiR408 and the involvement of them in modulating ABA signaling that possibly impacts on osmolytes accumulation, photosynthesis, and salt tolerance of plants upon salt stress.

**FIGURE 9:**
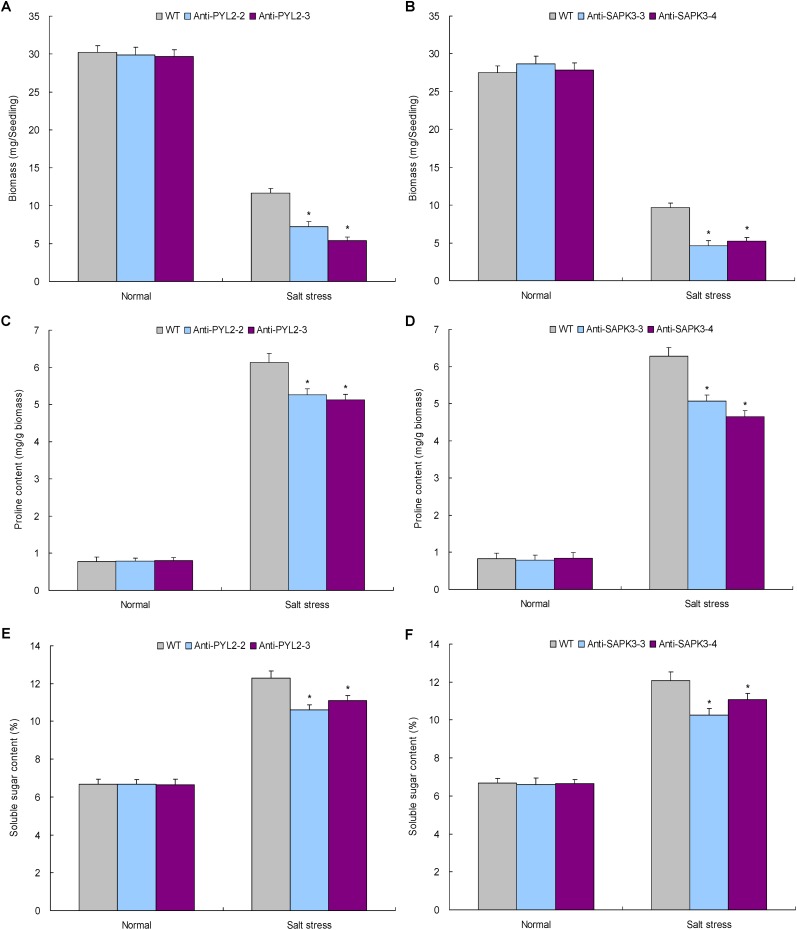
The biomass, proline contents, and the soluble sugar contents in the lines with *NtPYL2* and *NtSAPK3* knockdown expression under the salt stress treatment. **(A**,**B)** The biomass; **(C,D)** the proline content; **(E,F)**, the soluble sugar content. Anti-PYL2-2 and Anti-PYL2-3, two lines with *NtPYL2* knockdown expression; Anti-SAPK3-3 and Anti-SAPK3-4, two lines with *NtSAPK3* knockdown expression; WT, wild type. Data are shown by average plus standard error and ^∗^ indicates to be statistically significant compared with WT (*P* < 0.05).

The function of *NtPYL2* and *NtSAPK3* in mediating salt response was investigated using transgenic lines with knockdown of these genes. Expression analysis revealed that the lines Anti-PYL2-2 and Anti-PYL2-3 showed drastic downregulation of *NtPYL2* whereas Anti-SAPK3-3 and Anti-SAPK3-4 repressed expression of *NtSAPK3* (**Supplementary Figures S5**, **S6**). Therefore, these lines together with WT cultured under normal condition or salt stress were subjected to evaluation of the gene function in mediating plant salt response. Under normal growth condition, all these lines exhibited comparable phenotypes, biomass, and proline and soluble sugar contents with WT seedlings (**Figures 8C,E**, **9A–F**). Under salt stress treatment, however, the lines displayed dramatically modified growth and osmolytes contents, showing smaller stature (**Figures 8D,F**), lower biomass (**Figures 9A,B**), and reduced contents of proline (**Figures 9C,D**) and soluble sugar (**Figures 9E,F**) than those of WT. These results suggested that the TaemiR408-mediated salt tolerance closely associates the miRNA role in modulating ABA signaling pathway via transcriptionally regulating distinct ABA signaling genes.

## Discussion

Plant Pi starvation response cross-talks frequently with other signaling pathways, including sugars, phytohormones, and photosynthesis as well as the abiotic stress of high salinity (Franco-Zorrilla et al., 2005; Rubio et al., 2009; Rouached et al., 2010; Baek et al., 2016). Fully understanding of the miRNA-mediated regulatory networks associating Pi starvation and salt responses can help elucidate the complex mechanisms as to how plants tolerate these stressors. A growing body of evidence has confirmed the critical role of miRNA-guided gene regulation in mediating plant response to diverse abiotic stresses, including Pi starvation and high salinity. To date, numerous miRNAs that are responses to Pi starvation (Fujii et al., 2005; Chiou et al., 2006; Zhao et al., 2013) and high salinity (Ding et al., 2009; Wang et al., 2014) have been identified. Based on transgene and mutants analyses, the essential function of distinct miRNAs in mediating Pi taken up (Fujii et al., 2005; Chiou et al., 2006) and internal Pi translocation (Bari et al., 2006), and modulating ABA signaling transduction (Baek et al., 2016) has been characterized. In this study, we characterized the function of wheat miR408, a conserved miRNA family member across diverse plant species involving responses to abiotic stresses, such as deprivations of copper (Ma et al., 2015), nitrogen and sulfur deficiency (Liang et al., 2015), and mediation of iron uptake (Paul et al., 2016) and drought tolerance (Hajyzadeh et al., 2015), in mediating plant adaptations to both Pi starvation and salt stress. Results indicated that the tobacco lines with TaemiR408 overexpression exhibited drastically improved phenotypes and biomass under the Pi starvation and salt stresses, which associate the miRNA role in elevating Pi acquisition, enhancing osmolytes biosynthesis, and improving photosynthesis behavior of plants upon these stressors. That TaemiR408 confers plants improved growth under both Pi starvation and salt stress suggests its value in genetically engineering crop cultivars with enhanced tolerance to these stressors. Additionally, TaemiR408 and its tobacco paralog shares identical precursor each other, suggesting the conserved nature of miR408 in monocots and eudicots.

Transcription of miRNAs upon environmental cues is similar to that of mRNAs, involving action of a set of factors, including the Pol II recruitment to promoter region mediated by transcriptional co-activators (Kim et al., 2011), the interaction between distinct transcription-associated proteins with essential TATA box (Xie et al., 2005), and the *cis*-regulatory motifs situated in miRNA promoters (Megraw et al., 2006). The modified transcription of miRNA upon diverse signaling is controlled by specific activators, generally the TFs (Hajdarpašić and Ruggenthaler, 2012; Liang et al., 2012). For example, the transcriptional response of miR398 to low copper deprivation is regulated by a TF gene *SQUAMOSA PROMOTER BINDING PROTEIN-LIKE7* (Yamasaki et al., 2009) whereas the expression of miR172, a temperature-sensitive miRNA, is repressed by a TF gene *SHORT VEGATATIVE PHASE* (Cho et al., 2012). Upregulated expression of miR399f, a typical Pi starvation-responsive miRNA, is under the control of a MYB2 TF (Baek et al., 2013). In addition, *cis*-regulatory motifs in promoters are crucial in characterizing gene transcription upon the environmental signaling. Among these, CRE motifs (CCGCGT, CACGTGT, and AAGTCAA) are enriched in the promoters of salt and drought-responsive genes, controlling largely gene transcription through interaction with distinct bZIP TFs (Ma et al., 2012). Likewise, PIBS and PIBS-like elements are frequently overrepresented in a large set of the Pi starvation-responsive genes, involving the modulation of gene transcription upon low-Pi signaling (Schünmann et al., 2004). In this study, TaemiR408 exhibited responses to both Pi starvation and salt stress, whose expression is upregulated by these stressors whereas its stress-induced expression is restored by the recovery treatments, suggesting the modified transcription of this wheat miRNA under above stressors. Further characterization of distinct transcription-associated factors, such as the activators, *cis*-regulatory motifs, and the transcription repressors involving TaemiR408 transcription, can help elucidate the mechanisms underlying the miRNA responses to Pi starvation and salt stress.

miRNAs-mediated stress response is closely associated with the target TF genes that transcriptionally regulate downstream stress-responsive or -defensive genes (Palatnik et al., 2003). In this study, six genes targeted by TaemiR408 are categorized into function families associating with biochemical metabolism, signaling transduction, and microtubule organization, rather than into the TF family. That TaMI408 does not target TF genes is possibly resulted from either the limited wheat cDNA database scanned against or preclusion of the miRNA targets from TF families. Further identification of the target genes can help establish the miR408/target action modules underlying this miRNA. The regulation of target genes under miRNA is accomplished mainly through cleavage or translation repression mechanisms (Mallory and Vaucheret, 2006). In this study, based on PPM-RACE and RLM-RACE, two approaches that effectively detect the target 3′ end fragments (i.e., *TaCP*, *TaMP*, *TaBCP*, and *TaFP*) and the target 5′ end fragments (i.e., *TaKRP* and *TaAMP*) after miRNA mediation, respectively, we confirmed the cleavage mechanism of target genes under regulation of this wheat miRNA member. Functional prediction analysis of these target genes revealed that they are involved in various processes, including biochemical metabolism, signaling transduction, and microtubule organization, suggesting their contribution to plant response to Pi starvation and salt stress possibly via diverse regulatory pathways.

Pi acquisition of plants is mediated by PT proteins during which ATP is served as the driving power. Plant PT proteins, such as the Arabidopsis PHT1 family (PHT1;1 to PHT1;9), share conserved function in mediating Pi uptake from media into root cells and regulating internal Pi translocation (Shin et al., 2004). miR399 mediates plant cellular Pi homeostasis to be associated with its transcriptional regulation of a set of PT genes, such as *Pht1;8* and *Pht1;9* (Bari et al., 2006; Chiou et al., 2006; Sunkar et al., 2012). In this study, investigation on P accumulation in TaemiR408 overexpression lines revealed the miRNA-improved Pi starvation tolerance associating with the enhanced Pi taken up. This finding indicated the possible connection between TaemiR408 and distinct PT genes. To address the PT genes underlying the regulation of this miRNA, we examined the expression patterns of six tobacco PT genes in the Pi-deprived TaemiR408 overexpresors and WT plants. Drastically upregulated expression of *NtPT2* in transgenic lines with respect to WT suggested its transcriptional regulation under miRNA and contribution to the TaemiR408-mediated enhancement of Pi accumulation. Using transgenic lines with TaemiR408 knockdown, we confirmed the function of this PT gene in regulating Pi acquisition of plants upon Pi starvation. Therefore, the improved low-Pi adaptation mediated by TaemiR408/target associates the transcriptional regulation of distinct PT genes, such as *NtPT2*. Further characterization of the transcriptional mechanism of *NtPT2* underlying miR408 can provide insights into the miRNA-mediated Pi acquisition.

Abscisic acid impacts largely on plant growth and development as well as mediates drastically plant response to drought and salt stresses (Fujii and Zhu, 2009). After induction upon osmotic stresses, ABA is sensed by the receptor proteins such as pyrabactin resistance 1 and pyrabactin resistance 1-like (PYL) (Ma et al., 2009; Park et al., 2009). Once bound by PYLs, the ABA-receptor complexes interact with clade A protein phosphatase type 2Cs (PP2Cs), which relieves the inhibition of PP2Cs on sucrose non-fermenting 1-related protein kinase 2s (SnRK2s). SnRK2s thus further transmit the ABA signaling via phosphorylating downstream TFs, such as ABA-responsive element-binding factors (ABFs), and ultimately regulate expression of the ABA-responsive genes (Furihata et al., 2006; Vlad et al., 2008; Sirichandra et al., 2010). Characterization of the mutants of ABA receptor and SnRK2 genes has validated the function of the ABA signaling genes in mediating drought and salt responses (Fujii and Zhu, 2009). In this study, the TaemiR408 overexpressors exhibited enhanced osmolyte contents and photosynthetic function under salt stress with respect to WT, suggesting that the TaemiR408-mediated salt tolerance associates with the ABA signaling pathways. To address this issue, we assessed the expression patterns of an array of tobacco genes encoding ABA receptors and SnRK2 proteins in the salt-stressed TaemiR408 overexpression lines. An ABA receptor gene *NtPYL2* and a SnRK2 gene *NtSAPK3* displayed upregulated expression in the TaemiR408 overexpressors after salt stress, suggesting their transcriptional regulation under TaemiR408 module and involvement in mediating salt response. Based on transgenic lines with knockdown of these ABA signaling genes, we validated their function in mediating plant salt tolerance. Thus, our results suggest that the improved salt tolerance mediated by TaemiR408 is associated with the miRNA modulation for ABA signaling through transcriptionally regulating ABA signaling genes *NtPYL2* and *NtSAPK3*. Further characterizing the transcriptional mechanism of the ABA signaling genes underlying TaemiR408 module can put insights into the miRNA-regulated salt response.

miR408 shows multiple roles in regulating plant response to abiotic stresses, as shown in this current study and previously reported (Ma et al., 2015). In Arabidopsis, transgene analyses on miR408 have indicated that overexpression of this miRNA member confers plants enhanced sensitivity to drought stress aside from its role in improving tolerance to salinity, cold and oxidative stress through reduced reactive oxygen species and induced transcription of antioxidative encoding genes. In this study, to characterize the potential functions of TaemiR408 in mediating plant drought stress response, we cultured transgenic tobacco lines with overexpression of this miRNA under water deficit condition. Results indicated that the transgenic lines (OE2 and OE3) showed alleviated growth phenotype (**Supplementary Figure S7A**) and reduced biomass (**Supplementary Figure S7B**) relative to wild type after the drought treatment. This finding is in agreement with the miR408 function in Arabidopsis as aforementioned, suggesting the conserved role of this miRNA in mediating plant drought stress response. The molecular mechanism underlying how miR408 negatively regulates drought response needs to be further characterized.

Distinct miRNA members possess multiple functions in plant stress responses. For example, besides involvement in Pi signaling response, miR399f also mediates ABA responses, conferring plants improved salt and drought tolerance via transcriptionally regulating target genes *ABF3* and *CSP41b* (Baek et al., 2013). Constitutive expression of rice miR508 confers plants enhanced tolerance to both salt stress and N starvation in creeping bentgrass through modifying transcription of *AsAAO* and *COPPER ION BINDING PROTEIN1*(*AsCBP1*), in which, the miRNA-mediated salt response is accomplished mainly by influencing water retention, cell membrane integrity, chlorophyll content, cellular potassium homeostasis, and CATALASE (CAT) and ASCORBIC ACID OXIDASE (AAO) activity; the miRNA-modulated N starvation adaptation is brought about by regulating biomass production, N accumulation, chlorophyll synthesis, and NITRITE REDUCTASE (NiR) activity (Yuan et al., 2015). Similarly, TaMRR408 in this study showed dual roles in the mediation of abiotic stress responses, including Pi starvation and salt stress, via regulating biological processes associated with the Pi acquisition and the remodeling of ABA signaling, respectively. These discoveries suggest that miR408 is potential in genetically engineering crop cultivars with improved Pi use efficiency and salt stress tolerance.

## Author Contributions

KX and CG designed the research. QB, XW, XC, GS, and ZL conducted the experiment and performed the data analysis. KX wrote the paper. All authors contributed to the paper and approved the final manuscript.

## Conflict of Interest Statement

The authors declare that the research was conducted in the absence of any commercial or financial relationships that could be construed as a potential conflict of interest.

## Footnotes

1 www.mirbase.org

2 http://plantgrn.noble.org/psRNATarget/

3 http://plantgrn.noble.org/psRNATarget/

## Supplementary Material

FIGURE S1 Stem-loop structure of TaemiR408 and alignment result between TaemiR408 and NtMIR408. **(A)** Secondary stem-loop structure initiated by the TaemiR408 precursor. **(B)** Alignment result between TaemiR408 and its ortholog NtMIR408 in tobacco. In **(A)**, the TaemiR408 mature sequence is highlighted by pink color.

FIGURE S2 Target transcript levels and insertion copies in tobacco lines with TaemiR408 overexpression. **(A)** The TaemiR408 transcript levels in transgenic lines. **(B)** The TaemiR408 insertion copies in transgenic lines. OE1 to OE6, transgenic lines with TaemiR408 overexpression; WT, wild type. In **(A)**, data are normalized by internal standard and shown by average plus standard error and ^∗^ indicates to be statistically significant compared with WT (*P* < 0.05).

FIGURE S3 Expression patterns of the target genes in the TaemiR408 overexpression lines upon Pi-starvation and salt stresses. **(A)** Target gene *NtBCP*; **(B)** Target gene *NtKRP*; **(C)** target gene *NtABP.* OE2 and OE3, two lines with TaemiR408 overexpression; WT, wild type. Data are normalized by internal standards and shown by average plus standard error and ^∗^ indicates to be statistically significant compared with WT (*P* < 0.05).

FIGURE S4 Target gene transcripts in transgenic lines with knockdown of *NtPT2.* Anti-PT2-1 to Anti-PT2-5, transgenic lines with *NtPT2* knockdown; WT, wild type. Data are normalized by internal standard and shown by average plus standard error and ^∗^ indicates to be statistically significant compared with WT (*P* < 0.05).

FIGURE S5 Target gene transcripts in transgenic lines with knockdown of *NtPYL2.* Anti-PYL2-1 to Anti-NtPYL2-7, transgenic lines with *NtPYL2* knockdown; WT, wild type. Data are normalized by internal standard and shown by average plus standard error and ^∗^ indicates to be statistically significant compared with WT (*P* < 0.05).

FIGURE S6 Target gene transcripts in transgenic lines with knockdown of *NtSAPK.* Anti-SAPK3-1 to Anti-SAPK3-7, transgenic lines with k *NtSAPK3* knockdown; WT, wild type. Data are normalized by internal standard and shown by average plus standard error and ^∗^ indicates to be statistically significant compared with WT (*P* < 0.05).

FIGURE S7 Phenotype and biomass of transgenic lines overexpressing TaemiR408 under drought stress. **(A)** Phenotype; **(B)** biomass. OE2 and OE3, two lines with TaemiR408 overexpression; WT, wild type. In **(B)**, Data are shown by average plus standard error and ^∗^ indicates to be statistically significant compared with WT (*P* < 0.05). 10-day-old transgenic and WT seedlings were cultured in vermiculite and regularly supplied by standard MS solution (sustaining 60–85% of soil water content) for normal growth or half amount of MS solution (sustaining 45–55% of soil water content) for drought treatment. Three weeks later, the transgenic and WT plants were subjected to recordation of phenotype and assay of biomass.

TABLE S1 PCR primers used in this study.
