# Supplementary figures and images for: Wheat miRNA TaemiR408 Acts as an Essential Mediator in Plant Tolerance to Pi Deprivation and Salt Stress via Modulating Stress-Associated Physiological Processes
Source: Front Plant Sci. 2018 Apr 18;9:499. doi: 10.3389/fpls.2018.00499 (PMC5916090; doi:10.3389/fpls.2018.00499)

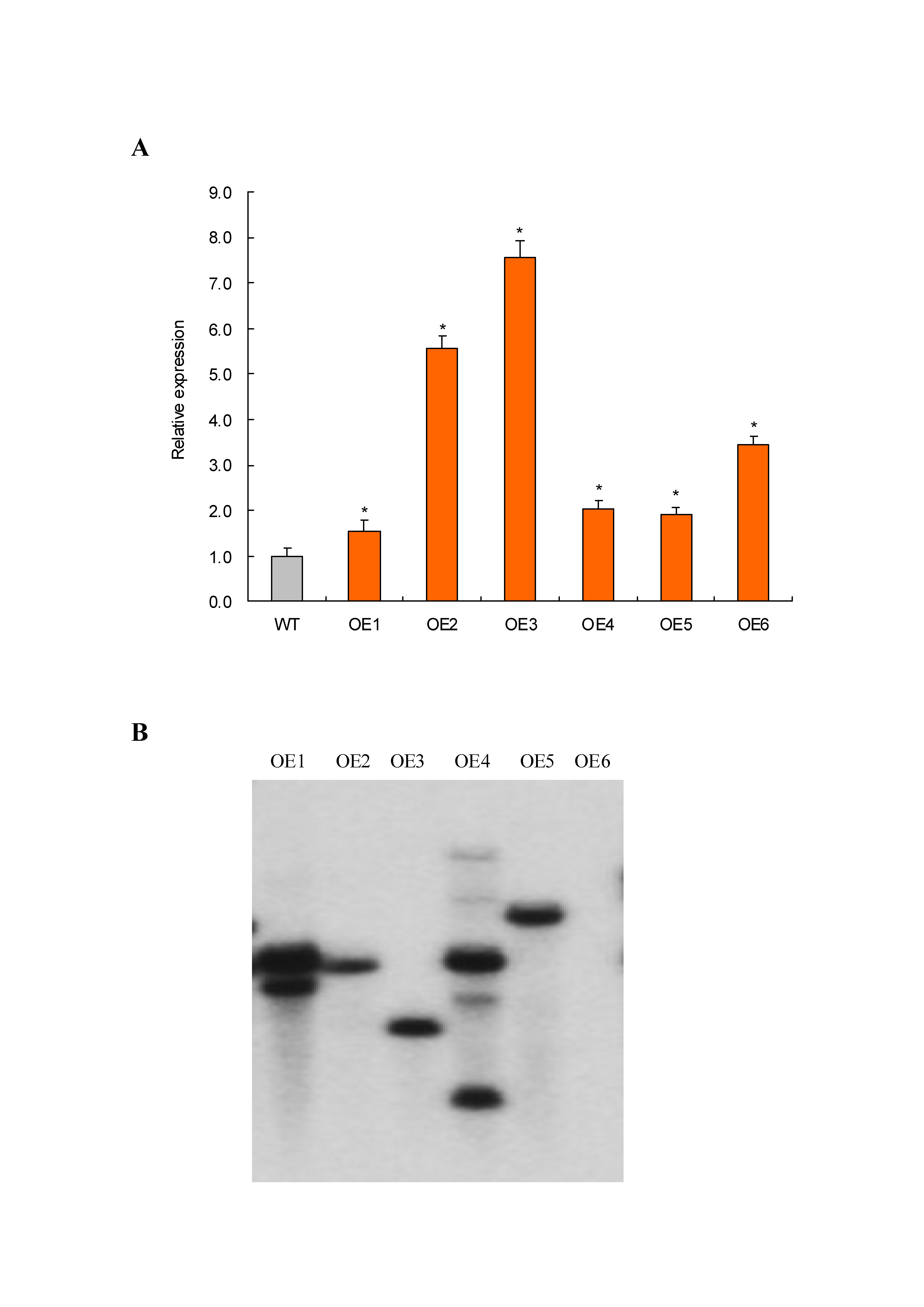

Supplement: FIGURE S2 — Target transcript levels and insertion copies in tobacco lines with TaemiR408 overexpression. (A) The TaemiR408 transcript levels in transgenic lines. (B) The TaemiR408 insertion copies in transgenic lines. OE1 to OE6, transgenic lines with TaemiR408 overexpression; WT, wild type. In (A), data are normalized by internal standard and shown by average plus standard error and ∗ indicates to be statistically significant compared with WT (P < 0.05). [file Image_2.TIF]

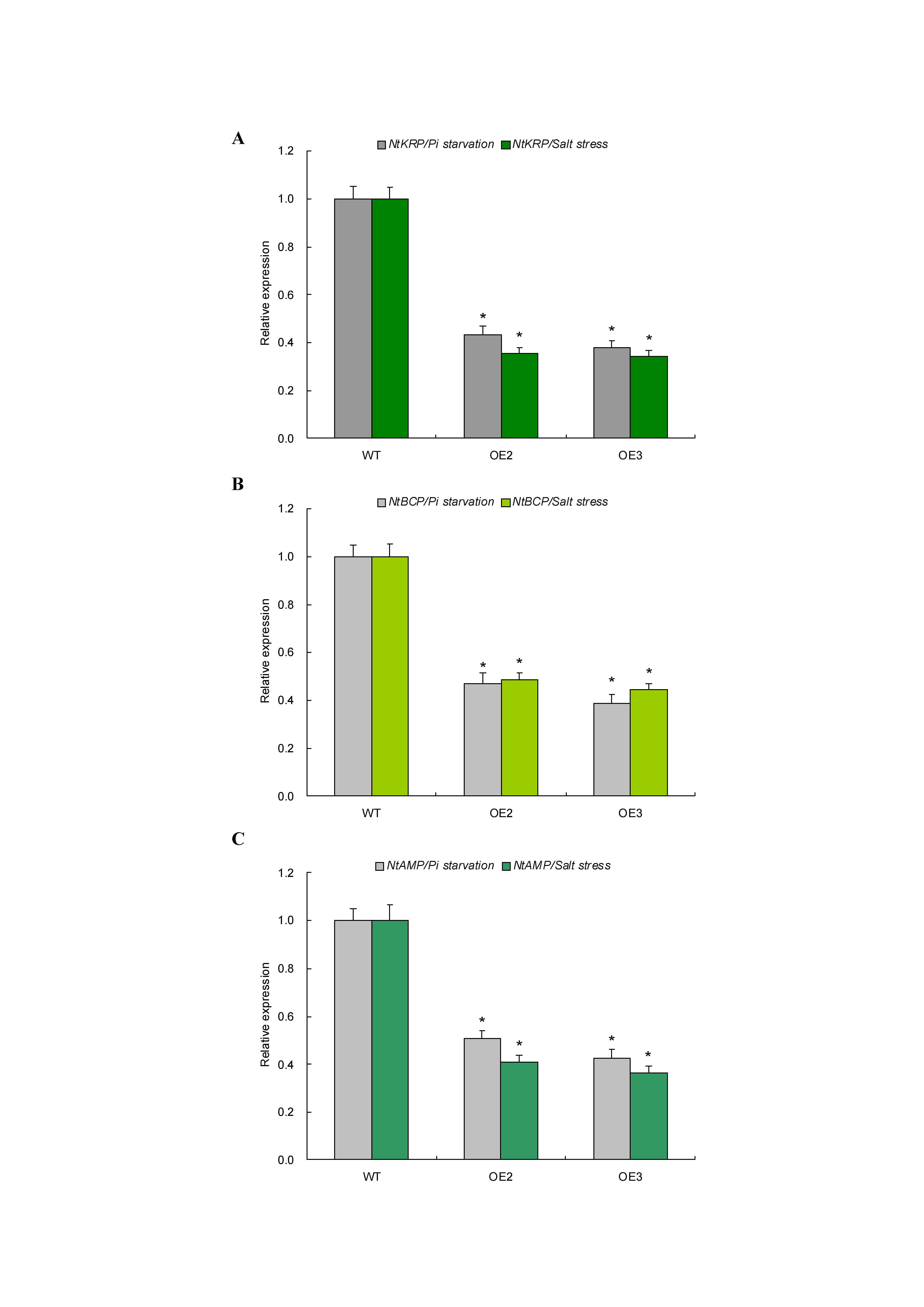

Supplement: FIGURE S3 — Expression patterns of the target genes in the TaemiR408 overexpression lines upon Pi-starvation and salt stresses. (A) Target gene NtBCP; (B) Target gene NtKRP; (C) target gene NtABP. OE2 and OE3, two lines with TaemiR408 overexpression; WT, wild type. Data are normalized by internal standards and shown by average plus standard error and ∗ indicates to be statistically significant compared with WT (P < 0.05). [file Image_3.TIF]

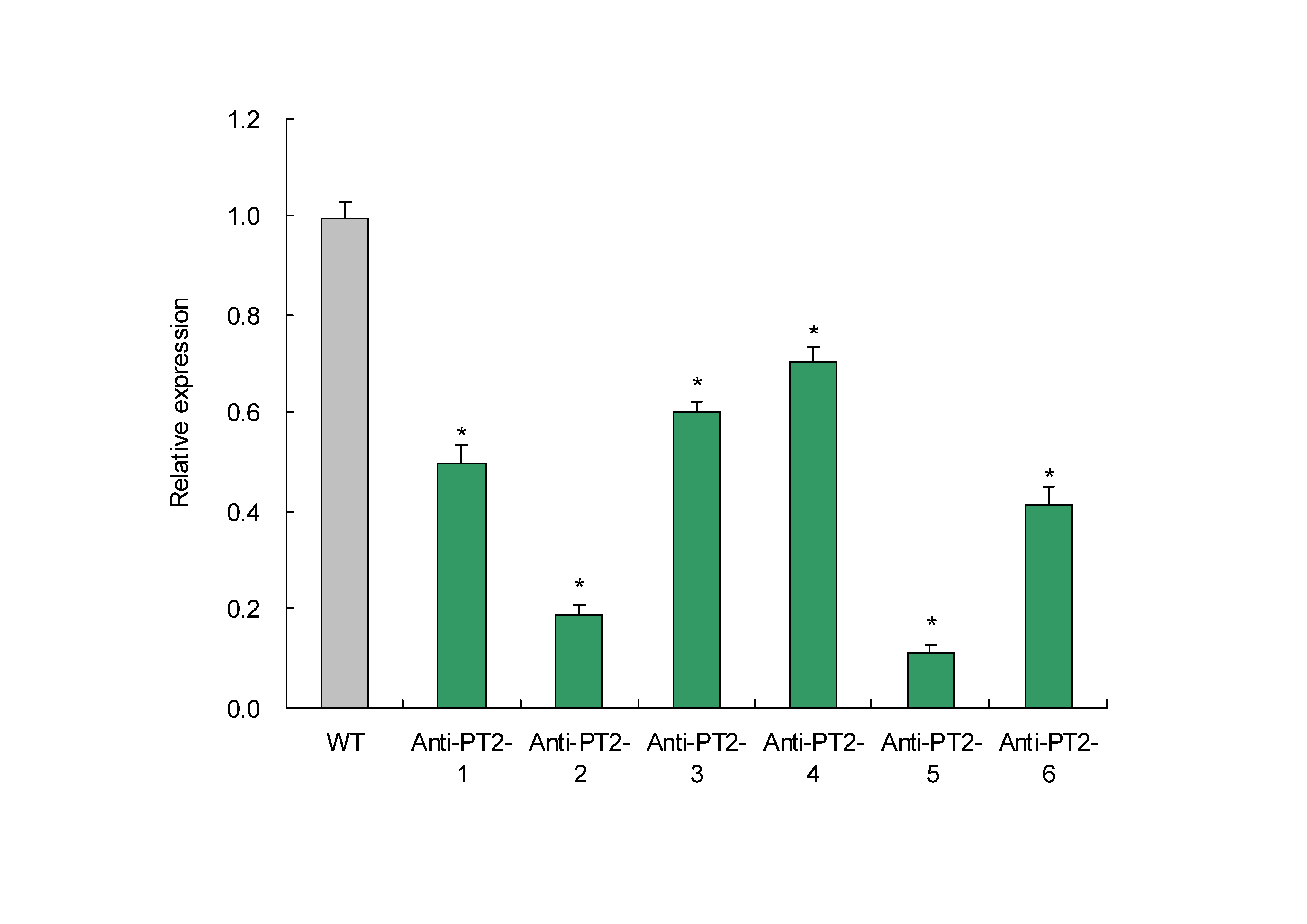

Supplement: FIGURE S4 — Target gene transcripts in transgenic lines with knockdown of NtPT2. Anti-PT2-1 to Anti-PT2-5, transgenic lines with NtPT2 knockdown; WT, wild type. Data are normalized by internal standard and shown by average plus standard error and ∗ indicates to be statistically significant compared with WT (P < 0.05). [file Image_4.TIF]

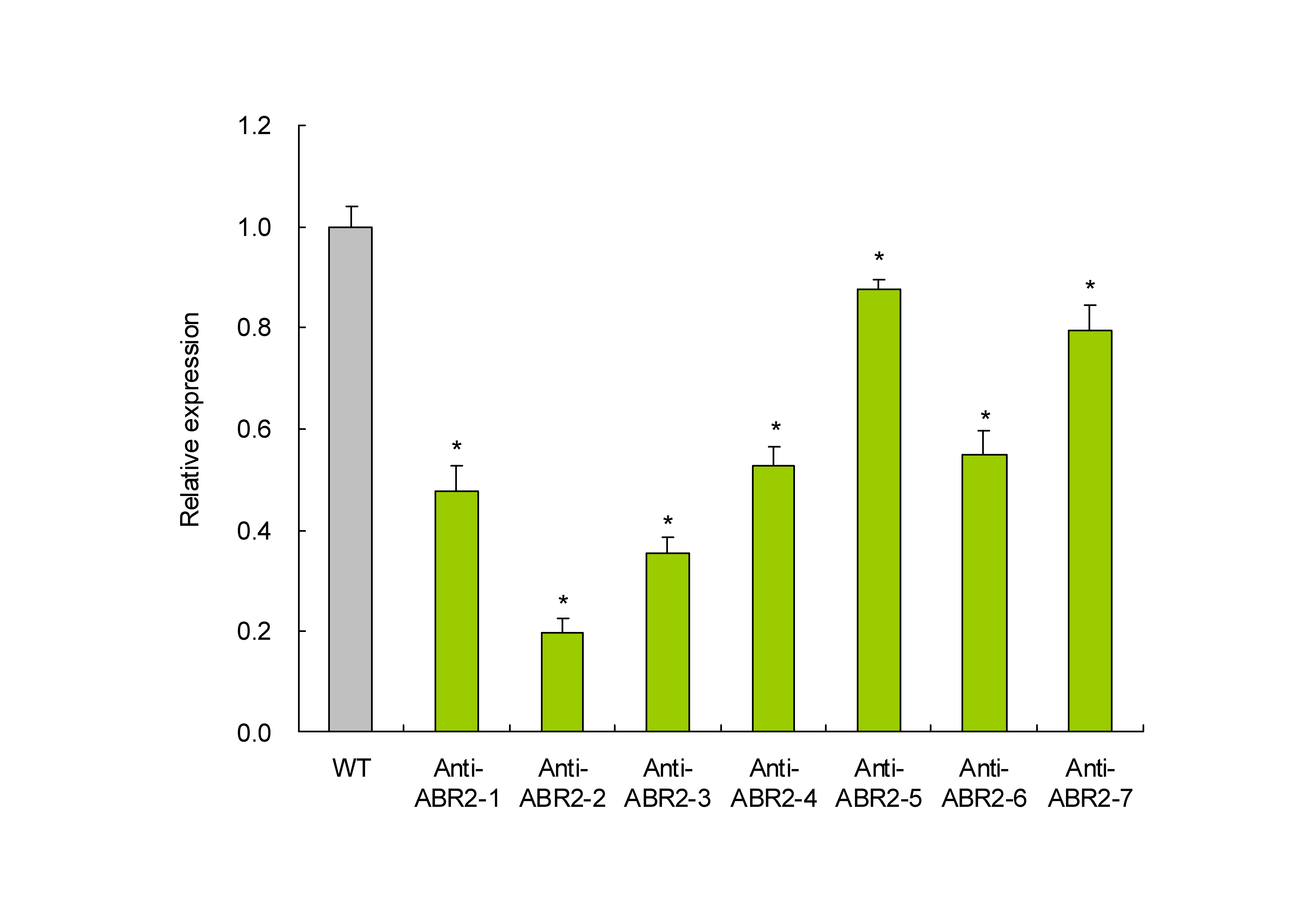

Supplement: FIGURE S5 — Target gene transcripts in transgenic lines with knockdown of NtPYL2. Anti-PYL2-1 to Anti-NtPYL2-7, transgenic lines with NtPYL2 knockdown; WT, wild type. Data are normalized by internal standard and shown by average plus standard error and ∗ indicates to be statistically significant compared with WT (P < 0.05). [file Image_5.TIF]

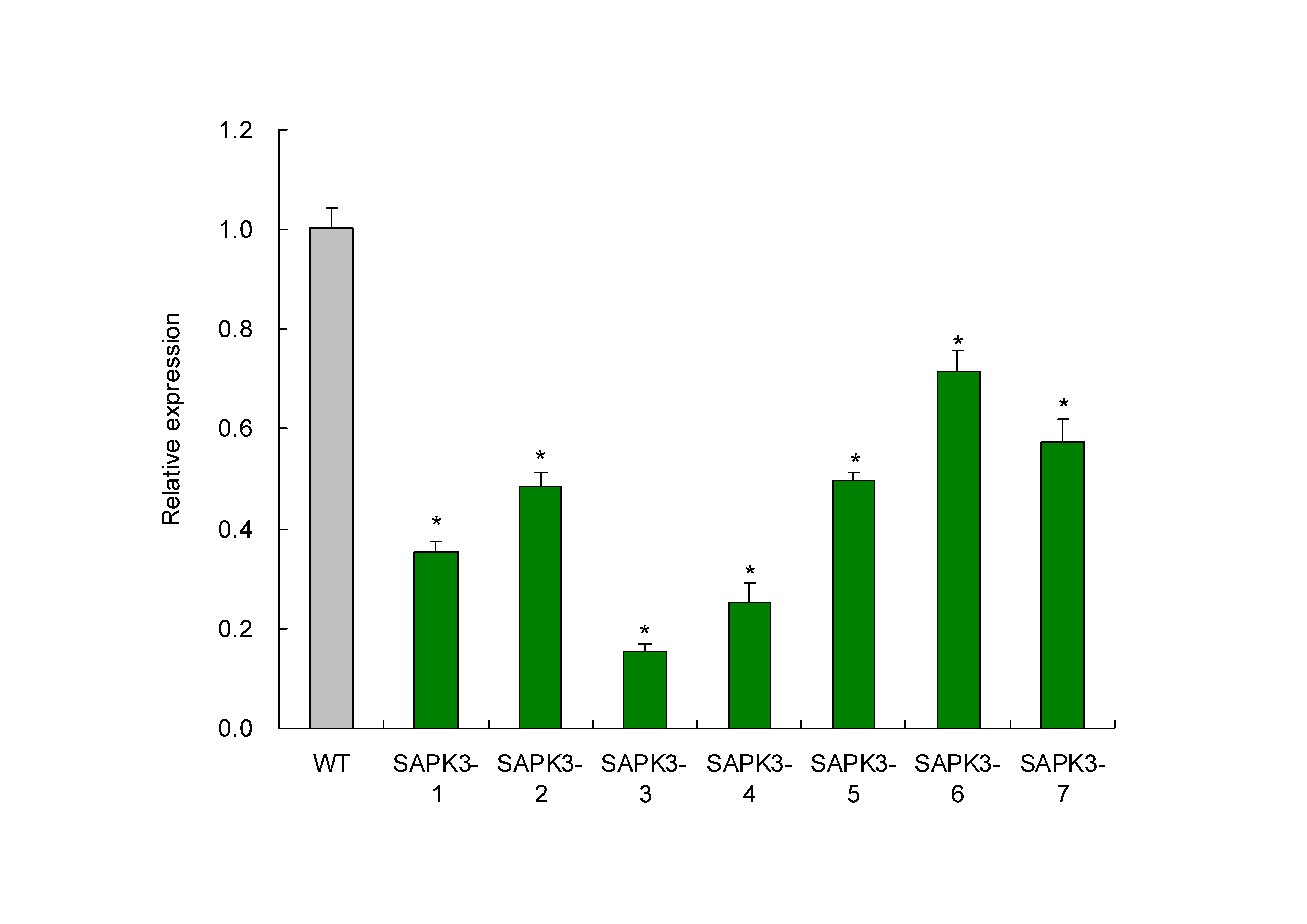

Supplement: FIGURE S6 — Target gene transcripts in transgenic lines with knockdown of NtSAPK. Anti-SAPK3-1 to Anti-SAPK3-7, transgenic lines with k NtSAPK3 knockdown; WT, wild type. Data are normalized by internal standard and shown by average plus standard error and ∗ indicates to be statistically significant compared with WT (P < 0.05). [file Image_6.TIF]

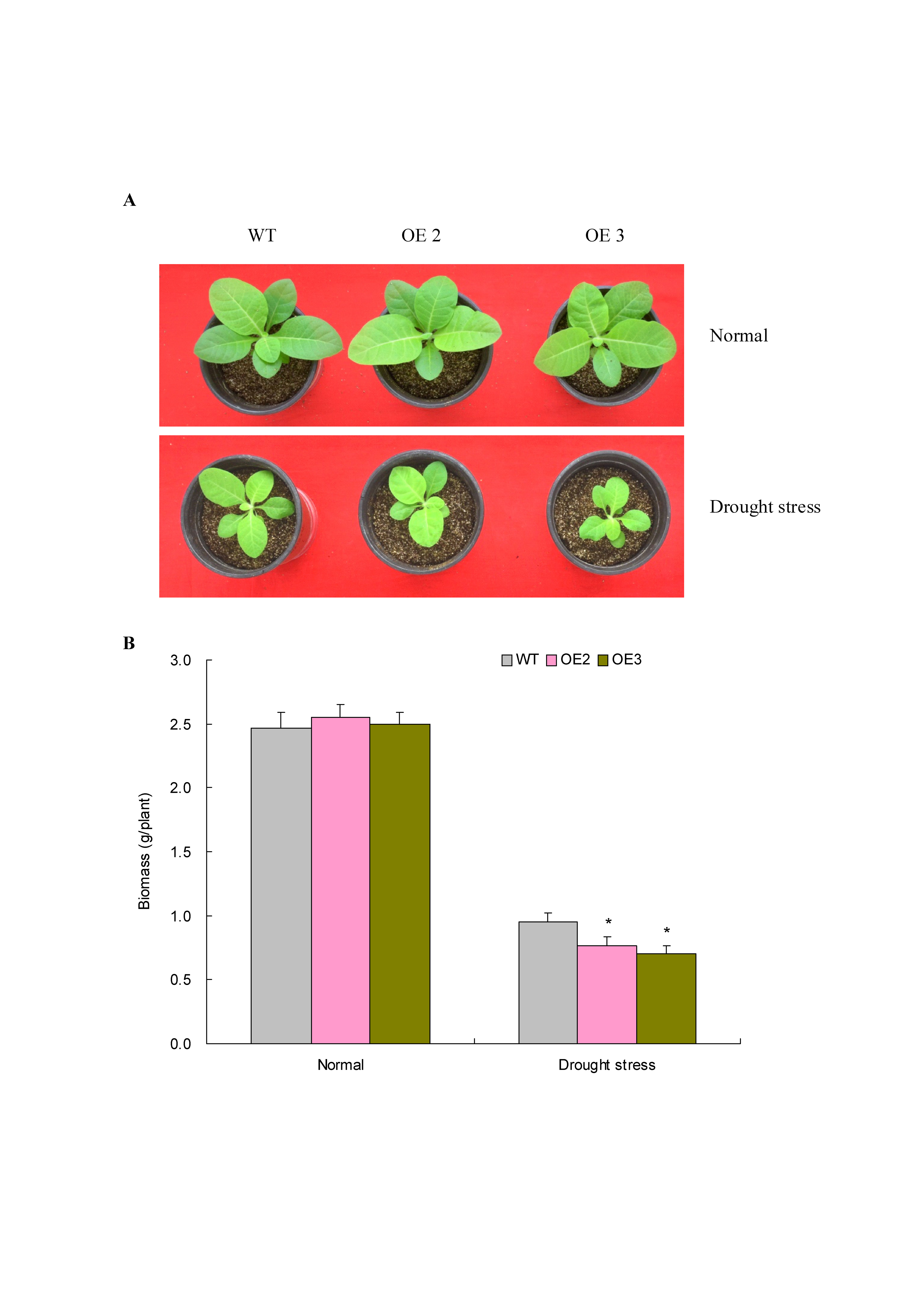

Supplement: FIGURE S7 — Phenotype and biomass of transgenic lines overexpressing TaemiR408 under drought stress. (A) Phenotype; (B) biomass. OE2 and OE3, two lines with TaemiR408 overexpression; WT, wild type. In (B), Data are shown by average plus standard error and ∗ indicates to be statistically significant compared with WT (P < 0.05). 10-day-old transgenic and WT seedlings were cultured in vermiculite and regularly supplied by standard MS solution (sustaining 60–85% of soil water content) for normal growth or half amount of MS solution (sustaining 45–55% of soil water content) for drought treatment. Three weeks later, the transgenic and WT plants were subjected to recordation of phenotype and assay of biomass. [file Image_7.TIF]
