# Supplementary material for: Wheat miRNA TaemiR408 Acts as an Essential Mediator in Plant Tolerance to Pi Deprivation and Salt Stress via Modulating Stress-Associated Physiological Processes
Source: Front Plant Sci. 2018 Apr 18;9:499. doi: 10.3389/fpls.2018.00499 (PMC5916090; doi:10.3389/fpls.2018.00499)
Supplement: TABLE S1 — PCR primers used in this study. [file Table_1.DOC]

**Table S1.** PCR primers used in this study

| **Purpose** | **Accession number** | **Forward primer (5´-)** | **Reverse primer (5´-)** |
| --- | --- | --- | --- |
| TaemiR408 expression | MI0006177 | ATTTTGTGAGTGGAGAGGGG | AGAGAGGGGGAGGGAGAGATT |
| *Tatubulin* expression | U76558 | CATGCTATCCCTCGTCTCGACCT | CGCACTTCATGATGGAGTTGTAT |
| *Nttubulin* expression | U91563 | TACACAGGGGAAGGAATGG | CTCGAAACCAACGGTATC |
| *TaCP* expression | KC852069 | TTCCACACACCTTTTGGCCTAC | AACAGTACACATTGATCGAGGAC |
| *TaMP* expression | XM_020292436 | TCGACTACTCCGGCTGGACC | CGCACAATCCATAATGCTCTGG |
| *TaBCP* expression | XM_020294339 | TCAAAGTCGGCGACACCCTCGT | CAACAACCGATCGTGACATGTTT |
| *TaFP* expression | XM_020310872 | AAGGAGCTTGCTAGTGCAGCC | AGTAGAAACCGCACCAAACCAC |
| *TaKRP* expression | XM_020290582 | TGCACGGAGATTGCTTCTCGAG | TAGAGTTGACATCTCAGCATCGAT |
| *TaAMP* expression | BJ225979 | TCTGTAGCCTTCCTCCCATAAGC | AGTTGCAAACACTCTTGAAGGC |
| *TaCP* 3**´** RACE | KC852069 | GGACACTGACATGGACTGAAGGAGT | ATTGATCGAGGACTTGTGAAAG |
| *TaMP* 3**´**RACE | XM_020292436 | GGACACTGACATGGACTGAAGGAGT | ACAATCCATAATGCTCTGGCAC |
| *TaBCP* 3**´**RACE | XM_020294339 | GGACACTGACATGGACTGAAGGAGT | CAACCGATCGTGACATGTTTCAT |
| *TaFP* 3**´**RACE | XM_020310872 | GGACACTGACATGGACTGAAGGAGT | AGAAACCGCACCAAACCACCAT |
| *TaKRP* 5**´**RACE | XM_020290582 | TTGGCGGACGGACTGGAGATGA | ATTCTAGAGGCCGAGGCGGCCGACATG |
| *TaAMP* 5**´**RACE | BJ225979 | TTCGGCACGAGGGAAGAGGCAG | ATTCTAGAGGCCGAGGCGGCCGACATG |
| NtMIR408 overexpression cassette | MI0006177 | AAACCATGGATTTTGTGAGTGGAGAGG | AAAGGTAACCAGAGAGGGGGAGGGAGAG |
| *NtBCP* expression | TC142445 | GTAAGATCATGTCTGGTCAGGG | TCATATAGGAAGTCTGATTAGAGC |
| *NtKRP* expression | FS391808 | GCGAAGCACCACTCTGCTAAAGAA | ACATAAACACGACCATACTCGCA |
| *NtAMP* expression | TC145853 | AACAAAAAGCAACCTTGAACGGG | TGTATTCATGCTCTCACCTACGC |
| *NtPT* expression | DI040486 | GCCATTCCATATCATCATTG | GACCTCATTTCTCTTCCCAC |
| *NtPT1* expression | AB020061 | GTTTACAGAATTGCAAGGGCGC | TTTTCCTTTAGATTCTGGCACC |
| *NtPT2* expression | AF156696 | CCATGGACTTCACTTGCTTG | ATTTCTCTTCCCACTTTCCC |
| *NtPT3* expression | AB042950 | GGTTCGTGATCATGTACTCA | GACCTCATTTCTCTTCCCAC |
| *NtPT4* expression | AB042951 | AGGGTACTGGTTCACCGTG | ATTATAATTACACACTTGGTCG |
| *NtPT5* expression | AB042956 | GCACTACTTAGTACAGTGCC | GCATCACAAAGTCTACCTCC |
| *NtPT2* knockdown expression cassette | AF156696 | TTTCCATGGATTCTGGCACCAAAAATG | TTTGGTAACCTTTTCCAAAAGGACATT |
| *NtPYR1* expression | XM_009778559 | ATGGAGCAATCTGAGAGCTC | CCTTGCTGTATCGTCTTTTCA |
| *NtPYL2* expression | XM_009778517 | TATGGCGGCCTCGGGGTGGT | TTGGTTACCTTCATTCAGCAGG |
| *NtPYL4* expression | XM_009780219 | ATGATGTACCAAGATTTCGGCC | TGTAGTAGACCAGTGTTATCGG |
| *NtPYL8* expression | XM_009788111 | GGACGACAGGTCTGTTTATGG | GAACGTTGTATTCCTTGGCGA |
| *NtPYL9* expression | XM_009771158 | GTCGGTGCCGTTAGTTTTTG | AGACACCCGCATATGCAGA |
| *NtPYL11* expression | XM_009596711 | GCCGAAGCAACGAGAGAC | ATCTAACAAGGTATTCGTCATG |
| *NtPYL12* expression | XM_009803914 | GCTCCACATGCATCCAATAA | AAGAAGTAATTAGTAGTATATG |
| *NtSAPK1* expression | XM_009765150 | ATAATGGAGCGTTATGAGTTTG | TTTGGGTAAGTTACAGCATTG |
| *NtSAPK2* expression | XM_009804544 | GGTTATTTGAGGTATTGAAGCT | CCGGTGTACAAAGCATCCT |
| *NtSAPK2;1* expression | XM_009768825 | GAATGGAAAGGTATGAAATTCTG | CTCATGCTTTAAGCAAATCGC |
| *NtSRK2A* expression | XM_009777772 | GAAGTTTGTGAAGGATAAGAAA | GACTAGGGGTTTGTCCACAG |
| *NtSRK2A;1* expression | XM_009775798 | GAAGTTAAGAAGGAGAGCTTT | CTCGGCTTCCGTTTGATCC |
| *NtSRK2E* expression | XM_009806284 | GATGGAGAATTACGAGCTGG | CTATGCTTTCTACAAGCAACAA |
| *NtSRK2E;1* expression | XM_009788259 | GGAGAGTTGTAGAGAAAGATA | TTCATCAACATGGCCTTGCG |
| *NtSRK2I* expression | XM_009778703 | CGAAATGGATCGAGGTCTGA | AAAGCCCCTTTCCAGGATAA |
| *NtSAPK3* expression | XM_009793723 | GGGTATAGAATGGAGGAAAA | TAACTAAGTCTTCTATACACC |
| *NtSAPK7* expression | XM_009782410 | GCAAGTAGGGTACAAGAATTA | TGTTGCCAAGTATTAGTTCCAC |
| *NtPYL2* knockdown expression cassette | XM_009778517 | TTTCCATGGAATARCAACAATGTAT | TTTGGTGACCTCCATATCAACCCC |
| *NtSAPK3* knockdown expression cassette | XM_009793723 | TTTCCATGGAATGCTCACCATCCA | TTTGGTCACCAAACATTATCAGGTTT |
